# Super liquid repellent surfaces for anti-foaming and froth management

**DOI:** 10.1038/s41467-021-25556-w

**Published:** 2021-09-09

**Authors:** William S. Y. Wong, Abhinav Naga, Lukas Hauer, Philipp Baumli, Hoimar Bauer, Katharina I. Hegner, Maria D’Acunzi, Anke Kaltbeitzel, Hans-Jürgen Butt, Doris Vollmer

**Affiliations:** Max Planck Institute for Polymer Research, Mainz, Germany

**Keywords:** Nanoparticles, Wetting

## Abstract

Wet and dry foams are prevalent in many industries, ranging from the food processing and commercial cosmetic sectors to industries such as chemical and oil-refining. Uncontrolled foaming results in product losses, equipment downtime or damage and cleanup costs. To speed up defoaming or enable anti-foaming, liquid oil or hydrophobic particles are usually added. However, such additives may need to be later separated and removed for environmental reasons and product quality. Here, we show that passive defoaming or active anti-foaming is possible simply by the interaction of foam with chemically or morphologically modified surfaces, of which the superamphiphobic variant exhibits superior performance. They significantly improve retraction of highly stable wet foams and prevention of growing dry foams, as quantified for beer and aqueous soap solution as model systems. Microscopic imaging reveals that amphiphobic nano-protrusions directly destabilize contacting foam bubbles, which can favorably vent through air gaps warranted by a Cassie wetting state. This mode of interfacial destabilization offers untapped potential for developing efficient, low-power and sustainable foam and froth management.

## Abstract (web summary)

Wong et al. demonstrate the efficacy of super-amphiphobic surfaces for in situ defoaming and inhibition of foam growth while handling aqueous solutions. Without the use of chemical additives, their passive approach suggests a facile alternative route to froth management in industrial processes.

## Introduction

Defoaming is the process of destabilizing existing foam while antifoaming aims to prevent the formation of foam^1–4^. This is achieved respectively, by depositing chemicals onto bulk foams or within the target liquid. These chemicals are also known as defoaming or antifoaming agents and include oils^4^, hydrocarbons or waxes^4,5^, microparticles^2,3,6,7^, or mixtures^1,8^ of these. The agents enhance the coalescence of foam bubbles by speeding up the disintegration of bubble films^3,9^. The effects of size, shape, and degrees of hydrophobicity of microparticles on defoaming have also been intensively studied to optimize defoaming^2,3^. However, hydrophobic particles quickly become inactivated by surface-active surfactants, which are always present in foams. To circumvent this, oils are used as carrier fluids to deliver hydrophobic particles directly to the thin film separating neighboring bubbles^5,6^. Alternatively, oils can also be used independently for defoaming^2,3,6^. Although efficient, oils and/or particles can be environmentally harmful while also altering the properties of the final product. Therefore, they might need to be removed afterward, requiring subsequent energy-intensive separation processes^1,5,6^. These standing issues make alternative approaches highly desirable.

Surprisingly, the consequences of liquid-repellent coatings on defoaming and antifoaming have not been explored. Here, we demonstrate that liquid-repellent coatings show excellent antifoaming properties. Their antifoaming potential has likely been underestimated because the typical contact area between foam and surface is small. This can be rectified by the use of liquid-repellent coatings on three-dimensional surfaces, thus enabling bulk interaction and defoaming.

In this work, we first show that superamphiphobic^10^ and liquid-infused^11^ surfaces, so termed liquid-repellent surfaces, can speed up defoaming. Liquid-infused surfaces are composed of a rough surface, which is infused with a lubricant. Although efficient in the short term, they suffer from depletion of lubricant after repeated use. Superamphiphobic surfaces consist of an amphiphobic, hierarchically rough surface which traps a layer of air^12–20^. We use the term air in the airgaps to distinguish it from gas in foam bubbles. Superamphiphobic surfaces show remarkable defoaming and antifoaming properties because surface protrusions destabilize and rupture contacting foam bubbles. Gases released from burst bubbles escape through these surfaces’ airgaps. This technique does not cause leaching and damage of the surfaces: thus preventing contamination of the target liquid’s composition. Superamphiphobic surfaces remain functionally stable (Supplementary Movie 1) over multiple cycles while demonstrating enhanced performance in both defoaming (50%) and antifoaming (larger than 100%) compared to controls.

## Results and discussion

### Bulk defoaming via liquid-repellent surfaces

**Fig. 1:**
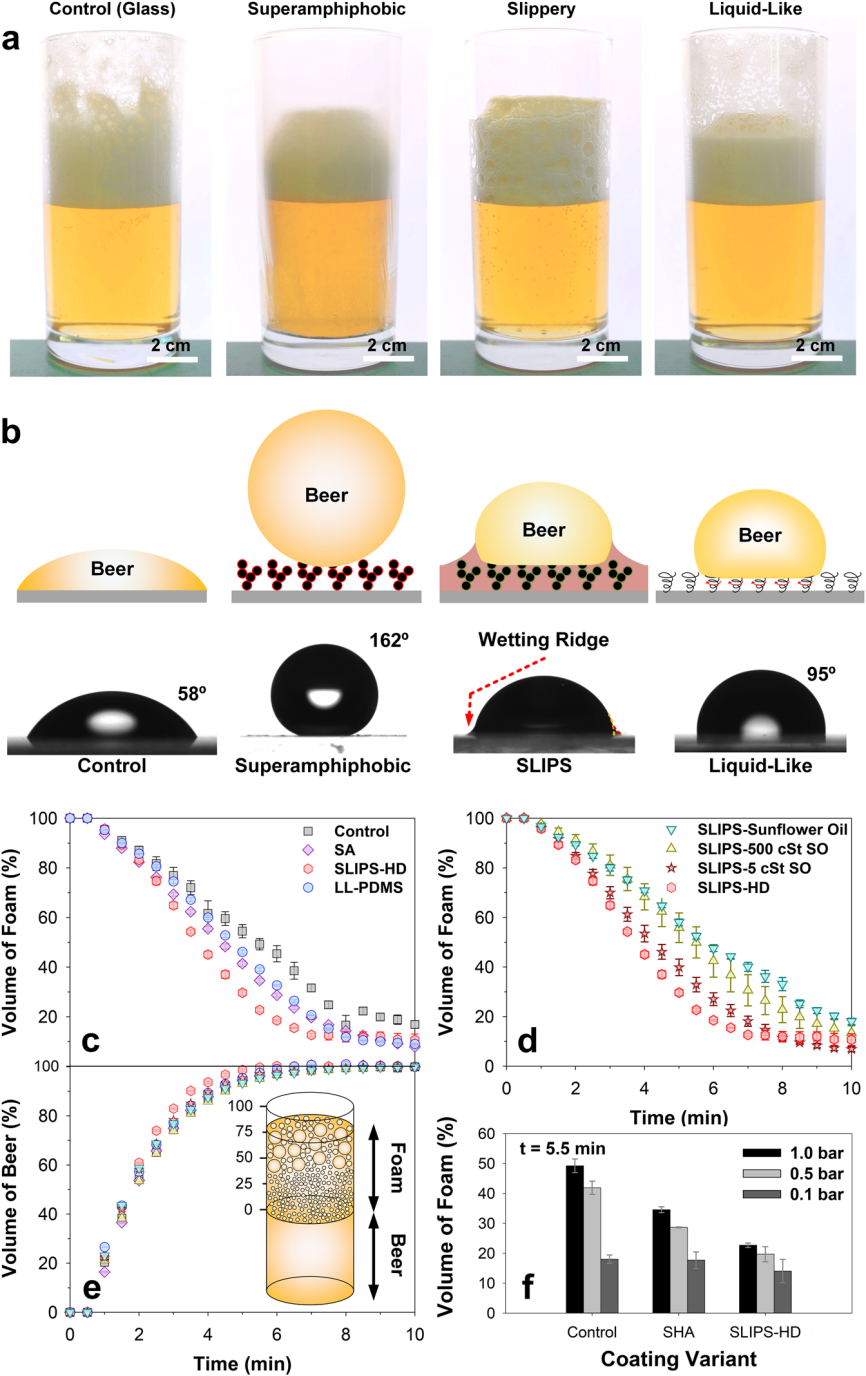
Evaluation of super liquid-repellent surfaces for defoaming. **a** Unmodified glass (control), superamphiphobic surface (SA), slippery liquid-infused porous surfaces (SLIPS), and liquid-like PDMS (LL-PDMS) 5 min after deposition. All surfaces were coated inside glass cups. As the wet liquid foam, commercially procured beer (Bitburger Pilsner) was used. **b** Schematic and optical image of a drop of beer (schematized as yellow) on test surfaces. The static, advancing, and receding contact angles were: (1) Control: 58°, 71°, 15°; (2) superamphiphobic: 164°, 180°^45^, 162°; (3) SLIPS: apparent contact angle of 74°, (4) liquid-like surface: 95°, 100°, 15°. Defoaming of foam generated from 1.0 bar pressure-dispensed beer. Analysis of (**c**) foam and (**e**) beer volume with test surfaces. **d** A comparison of various liquid-infused surfaces. **f** Volume of foam for three dispensing pressures using control, superamphiphobic and hexadecane-infused SLIPS glasses after 5.5 min. HD refers to hexadecane, SO refers to silicone oil. All data are presented in mean ± standard deviations.

Cylindrical glasses (internal diameter: 5.5 cm, height: 12 cm) were used as test surfaces for defoaming. Unfunctionalized soda-lime glass represents the control. The inner surfaces of glasses were modified by coating the walls with a superamphiphobic or a liquid-infused layer. The superamphiphobic surface (SA) was synthesized by depositing surface-functionalized nanoparticles^10^ onto a polystyrene binder (“Methods”). Superamphiphobicity was verified by low sliding angles (3° ± 1°) for hexadecane (*γ* = 27.5 mN m^−1^) drops (Supplementary Fig. 1). The slippery liquid-infused porous surface (SLIPS) variant comprises of a 2-µm-thick nanoparticle layer (Glaco) infused with various oils (“Methods”)^21^. SLIPS are also termed liquid-infused surfaces. Liquid-like surfaces were synthesized from PDMS brushes, liquid-like PDMS (LL-PDMS)^22^ (“Methods”). Photos of the foaming dynamics by beer foam in these glasses are represented in Fig. 1a. The wettability of a drop of beer on these surfaces greatly differs. The beer wets the glass control and contacts the other liquid-repellent surfaces, whereas a drop shows a high contact angle and easily rolls off a superamphiphobic coating (Fig. 1b and Supplementary Fig. 1).

Foamy beer was dispensed into the functionalized cups at dispensing pressures of 0.1–1.0 bar. We determined the initial height of the beer-foam mixture, ${H}_{{{{{{\rm{foam}}}}}}}\left(t=0\right)$, after filling the glasses. This height changes with time, as ${H}_{{{{{{\rm{foam}}}}}}}\left(t\right)$. After 10 min, the height of the liquid beer (without foam) remained constant within our experimental accuracy, defined as ${H}_{{{{{{\rm{beer}}}}}}}(\infty )$. The dynamic volume of foam, ${V}_{{{{{{\rm{f}}}}}}}$ is defined as,

1 $${V}_{{{{{{\rm{f}}}}}}}=\frac{{H}_{{{{{{\rm{foam}}}}}}}\left(t\right)-{H}_{{{{{{\rm{beer}}}}}}}({{\infty }})}{{H}_{{{{{{\rm{foam}}}}}}}\left(t=0\right)-{H}_{{{{{{\rm{beer}}}}}}}({{\infty }})}\times 100.$$

Analogously, we defined the volume of liquid beer, ${V}_{{{{{{\rm{b}}}}}}}$ as,

2 $${V}_{{{{{{\rm{b}}}}}}}=\frac{{H}_{{{{{{\rm{beer}}}}}}}\left(t\right)}{{H}_{{{{{{\rm{beer}}}}}}}({{\infty }})}\times 100.$$

The beer volume increases over time primarily because of the gravitational drainage of liquid from the foam^1^.

To increase contrast, backlighting (also termed shadowgraphy) was used. This made the foam column appear black (Supplementary Fig. 2 and Supplementary Movies 2–4). The height of the beer and the foam on the glass walls (contact lines) were computationally tracked using automated image processing techniques (Supplementary Fig. 2 and Supplementary Movie 5). Defoaming occurred fastest with SLIPS infused with hexadecane (Fig. 1c, SLIPS-HD, red hexagons). This was followed by SA (Fig. 1c, purple diamonds), LL-PDMS (Fig. 1c, red spheres), and the control (Fig. 1c, black squares). To test the influence of viscosity and interfacial tension, liquid-infused surfaces with silicone oils (SO, 5 cSt and 500 cSt) and sunflower oil were compared to hexadecane, which shows the fastest defoaming rate (Fig. 1d). Defoaming rates decreased with increasing viscosity. This is likely caused by the slower imbibition of oil into foams if the viscosity of oil increases. The presence of multicomponent impurities (sunflower oil) may also influence defoaming.

For comparison, we investigated the use of LL-PDMS brushes. However, they perform significantly worse at lower dispensing pressure (Supplementary Fig. 3). The lower dispensing pressure resulted in a foam head with comparatively lower stability despite a similar initial liquid fraction. With the use of liquid-like PDMS brushes, remnants are always left on the glass walls (Fig. 1a). The presence of persistent foam remnants disqualifies them from being the ideal surface variant for defoaming/antifoaming.

Figure 1f shows the foam volume at 5.5 min after dispensing. Superamphiphobic and liquid-infused glasses enhance defoaming at various dispensing pressures, albeit at different timescales (Supplementary Fig. 3). The foam volume remaining at 5.5 min decreased with decreasing dispensing pressure for all surfaces. At 1.0 bar, foam volume decreased by 15% for the superamphiphobic surface, relative to the control glass. For the liquid-infused glass, this difference was 25%. The gains in beer volume in the SLIPS-HD system (Fig. 1e, red hexagons) are marginally higher, complementing the fastest defoaming.

### Bubble size distribution and coalescence/rupture events

**Fig. 2:**
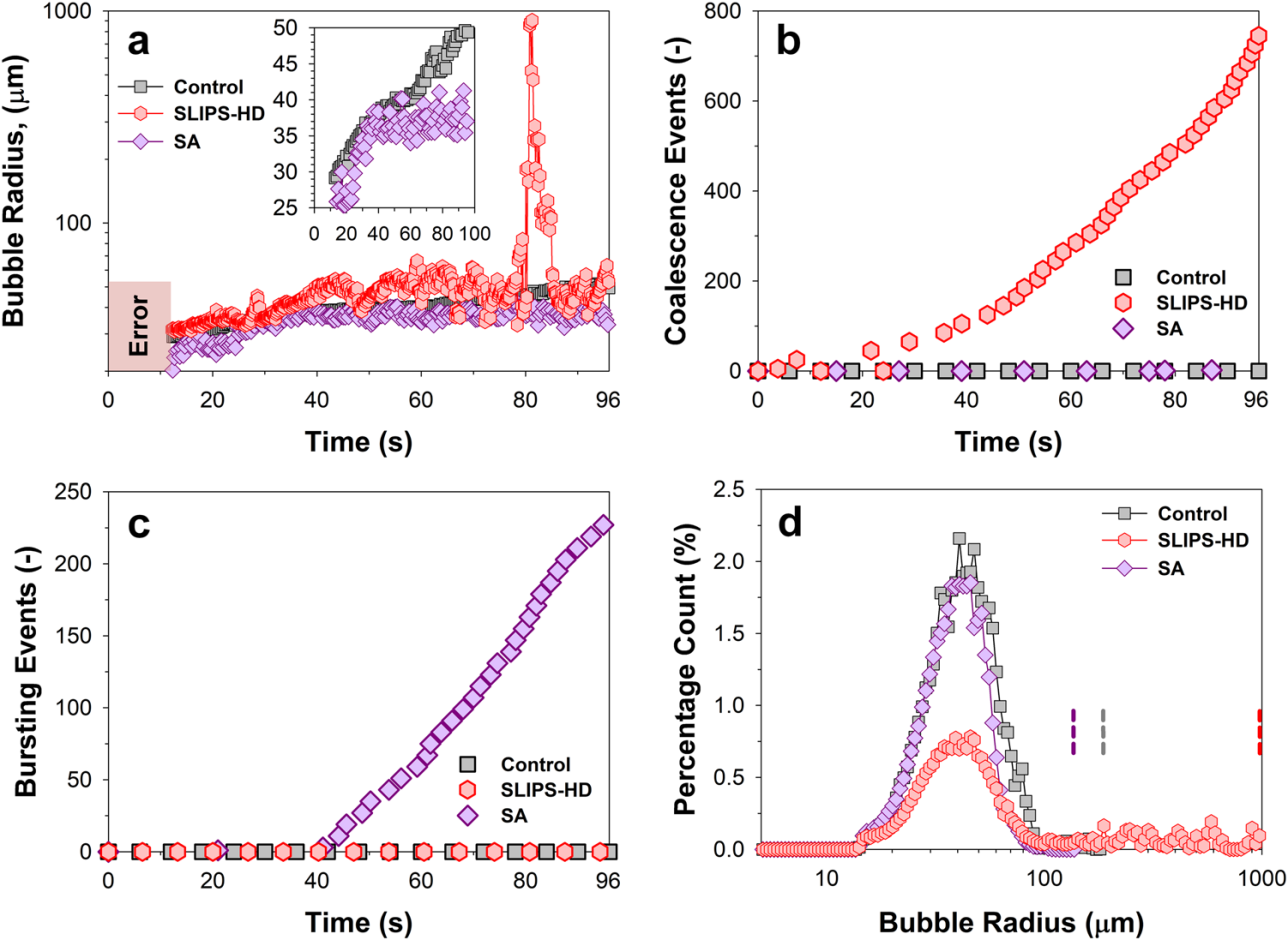
Bubble dimensions with respect to time. Imaging at 2.5 cm below the maximum original foam height. Bubbles were analyzed over a surface area of 3.5 mm^2^, at the bubble-to-surface interface. The foam line recedes from view beyond 96 s for the fastest defoaming superamphiphobic surface. Control refers to unfunctionalized glass, SLIPS-HD refers to a slippery liquid-infused porous surface infused with hexadecane, and SA refers to superamphiphobic glass. **a** Average bubble radii with respect to time, showing a gradual rise in the control (inset, gray squares). Bubbles remained smallest for the superamphiphobic system. **b** Coalescence events observed in SLIPS. **c** Bursting events caused by the dissipation of a bubble during bubble-to-surface interaction were observed for the superamphiphobic surface. Observation of the first bursting event is dependent on the region of observation. All bubbles were computationally tracked (see “Methods”). **d** Analysis of the percentage count of bubble radii and areas over the entire 96 s duration (minus the first 12 s) revealed persistently small bubbles in the control and superamphiphobic surfaces. The largest bubble radii in each system are represented with a dashed line.

To gain insight into the defoaming mechanisms, we determined the number and radii of bubbles in close contact with surfaces (Fig. 2 and Supplementary Fig. 4). At a dispensing pressure of 1.0 bar, the whole glass was filled with a foam having a liquid fraction, *ϕ* ≈ 0.45 at $t=0$. We imaged the formation and evolution of bubbles 2.5 cm below the top of the original foam head (investigated area ≈3.5 mm^2^). For detailed image analysis, we excluded the first 12 s because the small bubble radii and fast-dispensing motion blurred the images and led to poor automatization of image analysis (Videos M6 and M7). During drainage, liquid fraction in the foam can be verified by observing bubble geometries. Bubbles remain spherical and we did not observe jamming, hinting that the influence of osmotic pressure^23^ is small over the timespan of observations (see Supplementary Fig. 4 and Supplementary Movie 6).

In all systems, the average bubble radius, *R*, increased over time (Fig. 2a). In the control, bubble radii increased almost linearly with an average rate of approximately 0.3 µm s^−1^ (Fig. 2a, inset). Bubble growth is likely caused by the diffusion of CO_2_ from the beer into the bubbles^24^ because no coalescence events were detected in the control despite growth (Fig. 2b). Here, coalescence is defined as the merging of two originally separate bubbles through inter-bubble film rupture. For the superamphiphobic surface, bubble radii reached a maximum (*ca*. 37 ± 3 µm) at around 40 s and remained constant thereafter. Bubbles that are in contact with the superamphiphobic surface burst immediately and disappeared (Fig. 2c, purple diamonds and Supplementary Movie 6). Thus, superamphiphobic surfaces destabilize bubbles. Considering the total number of bursting events over time, a time delay of *ca*. 40 s was noted before a linear increase in bursting events occurs (Fig. 2c). During the first 40 s, we observed excess liquid flowing along the glass wall driven by gravitational drainage (Supplementary Movie 6 at 40 s)^25^. This liquid prevented bubbles from contacting the wall.

For SLIPS infused with low-viscosity hexadecane oil, a strongly fluctuating increase in bubble radii was observed. This is caused by the coexistence of a few large bubbles with a larger number of smaller bubbles after a few tens of seconds of contact (Supplementary Fig. 4). Bubbles interacting with a hexadecane-infused surface reached radii of up to 1 mm, as compared to the *ca*. 200 and 100 μm with the control and superamphiphobic surfaces, respectively (Fig. 2d). This is caused by the series of coalescence events that resulted in spontaneously large bubble sizes (Fig. 2a). These large singular bubbles can dominate the entire field of view, causing the peak in the evolution of bubble radii (Fig. 2a). However, they are quickly driven out of the field of view by buoyant forces, and replaced by relatively smaller bubbles (Fig. 2a–d). This is a universal observation for all oil-infused surfaces assessed, i.e., independent of viscosity and surface tension (Supplementary Movie 6, SLIPS variants).

To understand the defoaming mechanism, we should consider the mass transfer of CO_2_ in the wet liquid foam. CO_2_ diffuses from the liquid beer into the bubbles. Bubbles grow in size and their volumes increase. This occurs through two potential routes: (1) nucleation of smaller bubbles that diffuse into larger bubbles through Ostwald ripening. (2) Direct diffusion of gases from the liquid phase into the bubbles. The increasing volume presses bubbles in the foam head against the glass wall. Bubbles in close contact with the test surfaces can deform^26^.

### Defoaming mechanisms

**Fig. 3:**
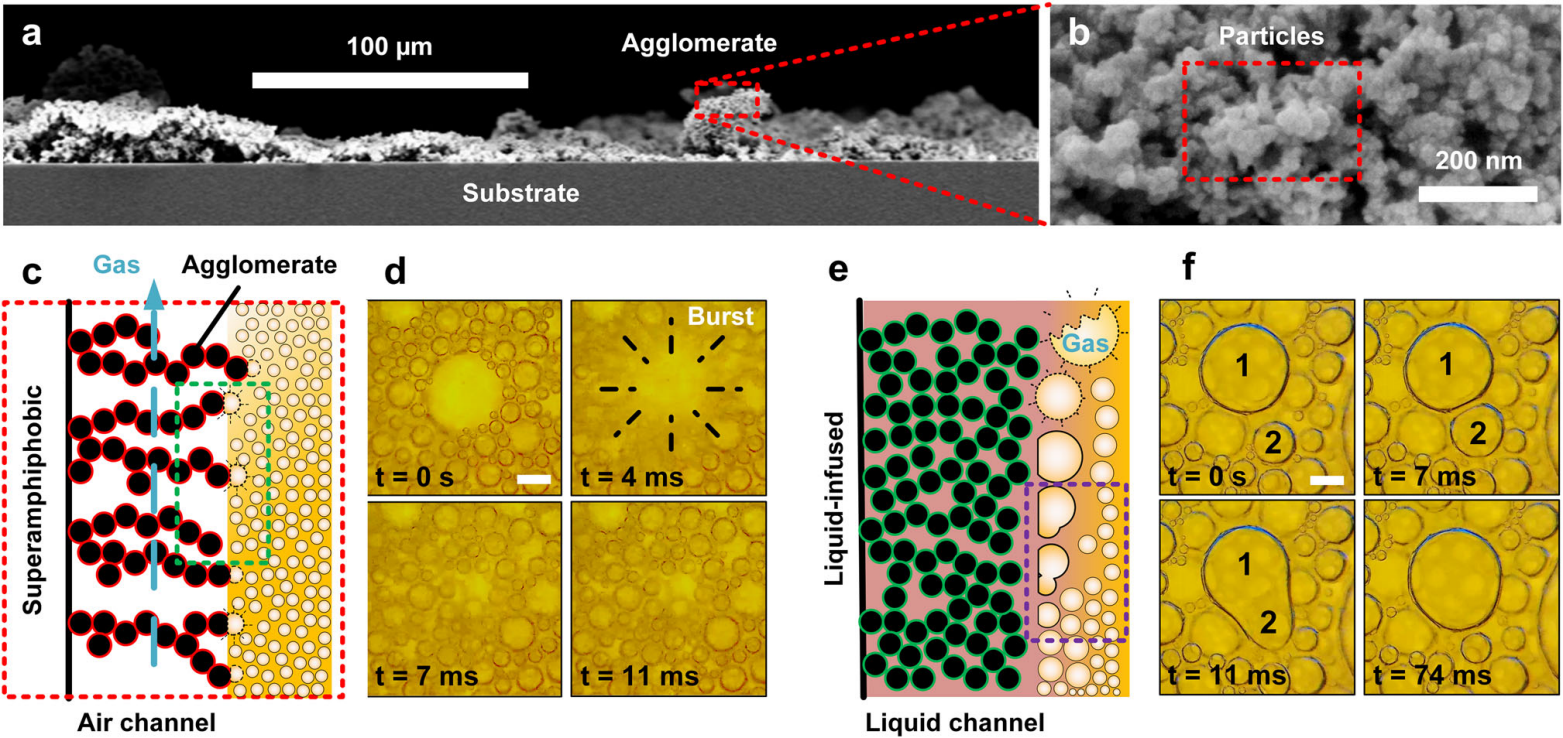
Foam decomposition by merging and bursting events. **a** Superamphiphobic coating (low-magnification side-profile) showing micro-agglomerates. **b** High-magnification image (top-profile) showing distinct nanoparticles (termed nanoprotrusions). **c** Sketch of a superamphiphobic surface. The bordered red particles depict functionalized agglomerates (fluorinated). **d** Macro-imaging of bubbles at the interface highlights a bursting event. Scale bar: 200 µm. A larger bursting bubble was chosen for image clarity. Bursting typically occurs in the range of bubble radii measured in Fig. 2. **e** Sketch of a liquid-infused surface. The green particles depict hydrophobized agglomerates (Glaco coating). The coating is infused with oil (light pink). **f** The foam bubble films (yellow) flatten close to the oil-infiltrated coating. Scale bar: 200 µm. Macro-imaging at the interface reveals a coalescence event of two bubbles (silicone-oil assisted). Schematics are not to scale.

For superamphiphobic surfaces, nanoprotrusions which are composed of nanoparticles (*ca*. 30 ± 10 nm diameter) stabilize the airgap (Fig. 3a, b). The bubble’s average radius (*ca*. 37 ± 3 µm) is much larger than the nanoprotrusions. When the bubble bursts, gases from the ruptured bubbles flow into the airgaps (Fig. 3c, d). The coating’s air channel transports and releases the gas into the ambient environment. This continuous replenishment of gas also helps to preserve the stability of the airgap/channel in the superamphiphobic layer.

In contrast, when a bubble makes contact with a liquid-infused surface, a wetting ridge forms (Fig. 3e, Supplementary Fig. 5 and Supplementary Discussion 2, Curvatures and Pressures in the Wetting Ridge*)*. As soon as the wetting ridges of neighboring bubbles overlap, bubbles experience a long-range attractive force (Supplementary Fig. 5 and Supplementary Movie 6, SLIPS)^27^. The attractive force depends on the size of both bubbles (Supplementary Fig. 5), the height of the wetting ridge, the interfacial tension, and the viscosity of the oil^28^. The bubbles merge upon contact with one another, increasing buoyancy (Fig. 3e, f). Coalesced bubbles move to the top of the foam column, thereafter destabilize and release trapped gases. When observed on a macroscopic scale, rapid decomposition of the foam column follows. After destabilization of the foam column, oil remains at the air–liquid interface. This reveals the unavoidable depletion of oil from within the slippery liquid-infused porous surface, hence limiting long-term performance (Supplementary Fig. 6).

Thus, superamphiphobic surfaces are more promising in the long run for extended operations and are hereafter further investigated. Defoaming in bulk foams stems from a combination of (1) gravitational drainage of liquid, (2) Ostwald ripening, (3) interfacial force-driven film thinning, and (4) spontaneous film rupture. Gravitational drainage typically requires several minutes before completion^25^. Previous simulation and experimental results reveal that the gravitational drainage of bulk wet foams is much slower compared to bubble film thinning from interfacial forces^29–31^. The dynamics and stability of bubbles in the close vicinity of the superamphiphobic layer depends mainly on interfacial forces between the solid–liquid interface at the top of protrusions and the liquid–gas interface of the bubble. Both may be coated by adsorbed layers of proteins or surfactants^31,32^.

**Fig. 4:**
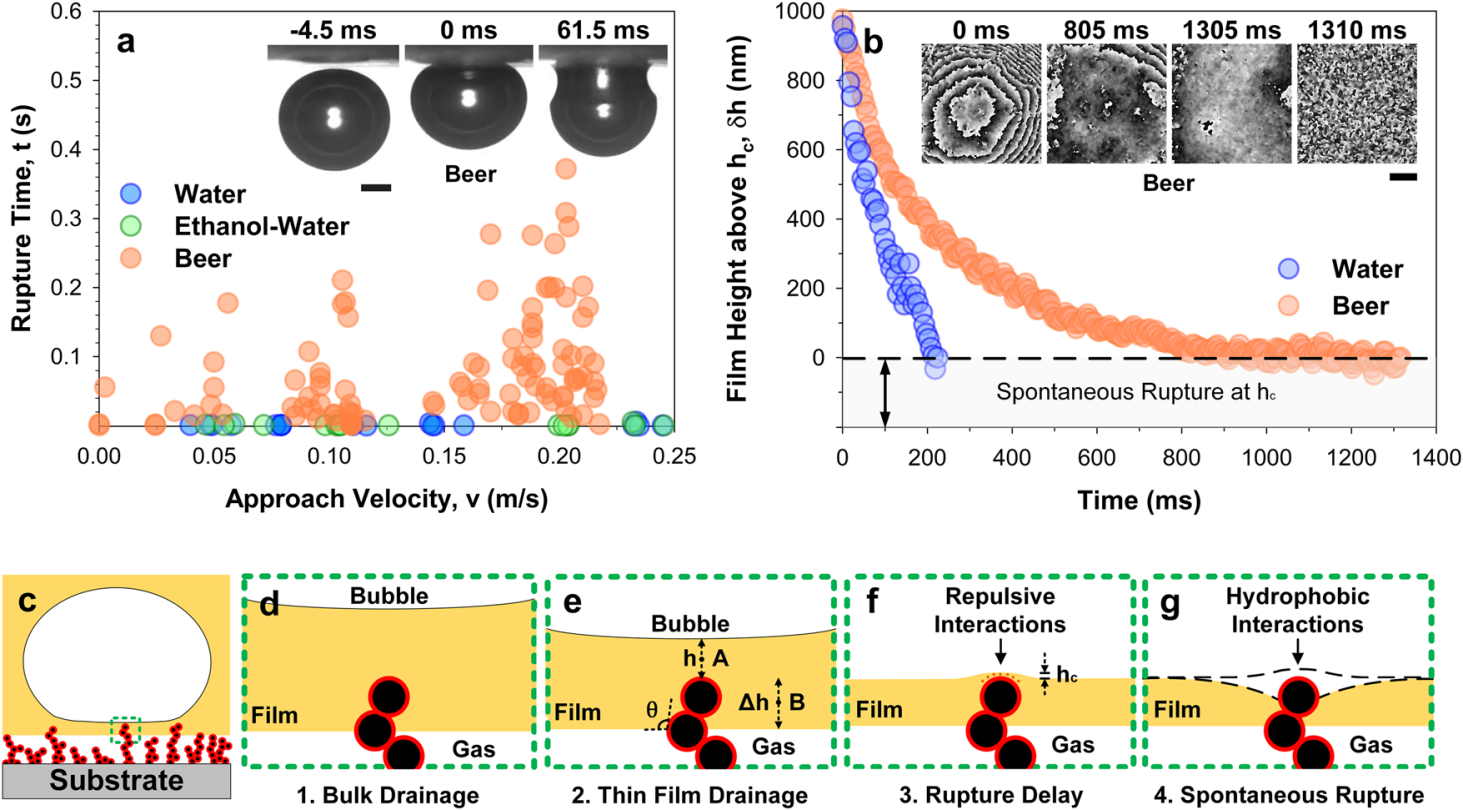
Mechanism of defoaming by bubble bursting. **a** Temporal analysis of bubble rupture using high-speed cameras. The approach velocities (bubble rise) varied between 1 and 25 cm s^−1^ using different release distances from the superamphiphobic surface. Liquids: water, ethanol–water mixture, and beer. Scale bar: 500 µm. **b** Spatial analysis of bubble rupture using holographic microscopy. The film height, $h\left(t\right)=\delta h\left(t\right)+{h}_{c}$, of a captive bubble during a slow controlled approach (5 µm s^−1^). The thickness of spontaneous rupture, ${h}_{c}$ is within the order of 100 nm for beer (orange circles) due to hydrophobic interactions. Inset: Phase maps monitoring the variation of film height. Height variation between dark and white lines is ≈ 300 nm. Scale bar: 100 µm. Schematics: **c** Approach of a bubble encountering a superamphiphobic surface. **d** Bulk drainage of liquid between the bubble and the nanoprotrusions. **e** Film thinning by interfacial and hydrodynamic forces. ${h}_{c}$, is the critical height of spontaneous rupture, taking into account the surface-penetrated depth determined using interference microscopy ($\triangle h$, Supplementary Fig. 7). **f** Momentary film stabilization due to repulsive interactions. **g** Hydrophobic interactions induce spontaneous rupture of a sufficiently thin film. Schematics are not to scale.

To gain insight into the timescales of bursting, we let single bubbles rise (buoyancy-driven), contacting a superamphiphobic surface. The bursting process was imaged with a high-speed camera (Fig. 4a) (Fastcam AX10, Photron, Japan). For deionized water (Fig. 4a, blue circles) and ethanol–water mixtures (Fig. 4a, green circles), bubbles burst within 1–10 ms after contacting the surface. In beer (Fig. 4a, orange circles), bubbles experienced a spread of rupture times of over three orders of magnitude (1 ms to 1 s) after making contact with the superamphiphobic surface. This delay in rupture timing suggests increased repulsive interactions in a protein-laden liquid as compared to water and ethanol–water mixtures.

To monitor film thinning with an improved spatial resolution (Fig. 4b), single bubbles encountering a superamphiphobic surface were analyzed using transmission holographic microscopy (T-DHM, T-1000, Lyncee-tec, Switzerland). In this case, we moved immobilized bubbles using a micromanipulator. Fixed bubbles approach the surface at 5 µm s^−1^ in both water (Fig. 4b, blue circles) and beer (Fig. 4b, orange circles). The shape of the bubble and the changing film height (or thickness), *δh*, were analyzed down to the point of film rupture (rupture height, ${{{{{{\rm{h}}}}}}}_{{{{{{\rm{c}}}}}}}$). In water, bubbles approach the surface without experiencing a slowdown (Fig. 4b, blue circles). Bubbles rupture within the time resolution of a single frame (5 ms).

In beer, the bubble deforms when approaching the superamphiphobic layer (Fig. 4b, inset: 0–805 ms). Deformation (deviation from spherical cap) reaches a maximum of ≈ 6 µm (Fig. 4b, orange circles, schematized Fig. 4c, d). The thin film was quasi-stable (Fig. 4b, orange circles, inset: 805–1305 ms) on the superamphiphobic surface for *ca*. 0.5 s before rupturing. Flattening of the interface is reflected in an increasing distance between interference fringes and the formation of an almost smooth area in the contact zone (Fig. 4b, inset: 805–1305 ms). The height varies by less than 200 nm. No dimple formation was noted. However, the intrinsic roughness of the superamphiphobic layer resulted in local changes in film thickness. Just before rupture, an agglomerate can be discerned. As observed in scanning electron micrographs in Fig. 3a, b, agglomerates are composed of nano- and micro-structured silica nanoparticles. This particular agglomerate is found, per the holographic image (Fig. 4b, inset: 1305 ms) in the bottom left quadrant as a black-colored distortion, indicative of a sharp change in profile. The scale of this distortion is *ca*. 50 μm in lateral dimensions. Rupture is likely initiated by the interaction of the thin film with the hierarchical profile.

Our current phenomenological observations allow for several possibilities on how exactly the rupturing may proceed. The corresponding timescale appears to be statistical, largely because information on flow boundary conditions and liquid rheology remains stochastic and incomplete. To start, beer contains ~4.5 wt% proteins which very likely populate the liquid–air interfaces^33^. The adsorption of a protein layer at the interface controls what happens during thin-film drainage. Two extremities bracket the situation:^34^ First, (1) no proteins assemble at the interface, mirroring the unique situation of pure water or water/ethanol mixtures^31,34^. Alternatively, (2) proteins pack densely at the interface, resulting in an effectively solid shell^35^. In reality, the assembly is stochastic in nature, and falls in between both extremes, creating an intermediate situation^35,36^. This is supported by the observations that while the film does not rupture immediately upon surface contact (per Case 1 as in pure liquids), it is also not indefinitely stable (per Case 2 as in stabilized foams). Continued film thinning is likely to facilitate improved packing of the protein layer^35^, moving the dynamically changing interface toward Case 2. When the thinning film is viewed from a continuum perspective after suitable coarse graining, the assignment of effective flow boundary conditions becomes rather difficult, as a rapidly changing rheology must be factored into the analysis, which is beyond the scope of this study. In Supplementary Discussion 4, Thin Film Behavior described by Stokes–Reynolds equation, we work out one scenario where no-slip boundary conditions are assumed to facilitate an analysis utilizing the Stokes–Reynolds equation. In this way, the magnitude of the timescale for film rupture (within the order of 100 ms) can be rationalized. Hydrodynamic effects are negligible because of the slow approach and the low radius of curvature of the protrusions.

The drainage of a bubble’s thin film near a superamphiphobic surface results from the pressure difference ($\varDelta P$) between the film at the protrusion (Fig. 4e, point A, film height $h(A)$) and the film away from the protrusion. $\varDelta P\approx$ $\varPi \left(h\right)$, where $\varPi (h)$ is the disjoining pressure due to interfacial forces. Away from the protrusion, we assume that $\varPi \left(h\right)$ (Fig. 4e, point B, film height ${h}_{c}$ + ${dh}$) is negligible since the film thickness is larger (excess depth, $\varDelta h$ = 350 ± 220 nm, Supplementary Fig. 7) than the effective range of interfacial forces^37^.

$\varPi \left(h\right)$ has contributions from van der Waals (${\varPi }_{{{{{{\rm{vdW}}}}}}}$) forces, electrical double layer (${\varPi }_{{{{{{\rm{EDL}}}}}}}$), and steric $({\varPi }_{{st}})$ forces due to adsorbed proteins. Disjoining pressure from van der Waals interactions $({\prod }_{{{{{{\rm{vdW}}}}}}}={A}_{{{{{{\rm{H}}}}}}}/6\pi {h}^{3})$ is the distance-dependent (*h*) parameter governing interactions between two phases (separating a medium) that can be repulsive (pulling liquid) or attractive (expelling liquid)^37^. The Hamaker constant (*A*_H_) may be approximated as fluoro–water–air, *A*_H_ = +1.6 × 10^−20^ J (attractive)^37^. In this instance, liquid films likely experience repulsive electrical double layer and attractive van der Waals forces^38,39^ once they have drained to thicknesses within the order of 10 and 100 nm^37^, respectively (Fig. 4c–e). However, attempts to model our experiments (Fig. 4b) without ${\varPi }_{{st}}$ failed (see Supplementary Discussion 4–6, Thin Film Behavior described by Stokes–Reynolds Equation). During film drainage of liquids such as beer, proteins likely adsorb at the interfaces (liquid–air, and liquid–fluoro). As we know that rupture eventually occurs, the adsorption is likely metastable^35^. During this quasi-stable equilibrium (Fig. 4b, orange circles, 900–1300 ms), adsorbed proteins may spontaneously move within the thin film. During this phase, steric repulsion by momentarily adsorbed proteins stabilizes the thin film (Fig. 4f). The film thickness hardly changes but rupture is delayed (Fig. 4b, orange circles).

During this time, spontaneous motion of the adsorbed proteins^35^ along and between the interfaces (liquid–air and liquid–fluoro) allows for stochastic time windows through which hydrophobic forces^40,41^ act (as they would immediately if the liquid was pure and clean, per Fig. 4b, blue circles). The mobility of any adsorbed proteins should be present to some extent, without which the thin film will experience indefinite stability. Once the hydrophobic forces momentarily act across the interface, the film ruptures and a three-phase contact is formed (Fig. 4f, g). Thereafter, the gases escape. The extent of remnant proteins adsorbed onto fluoro-functionalized nanoparticles is likely to be minimal, per previous anti-fouling studies using bovine serum albumin (BSA)^42^. Moreover, they may also be easily washed away during future interactions (with water or with more foam liquids).

**Fig. 5:**
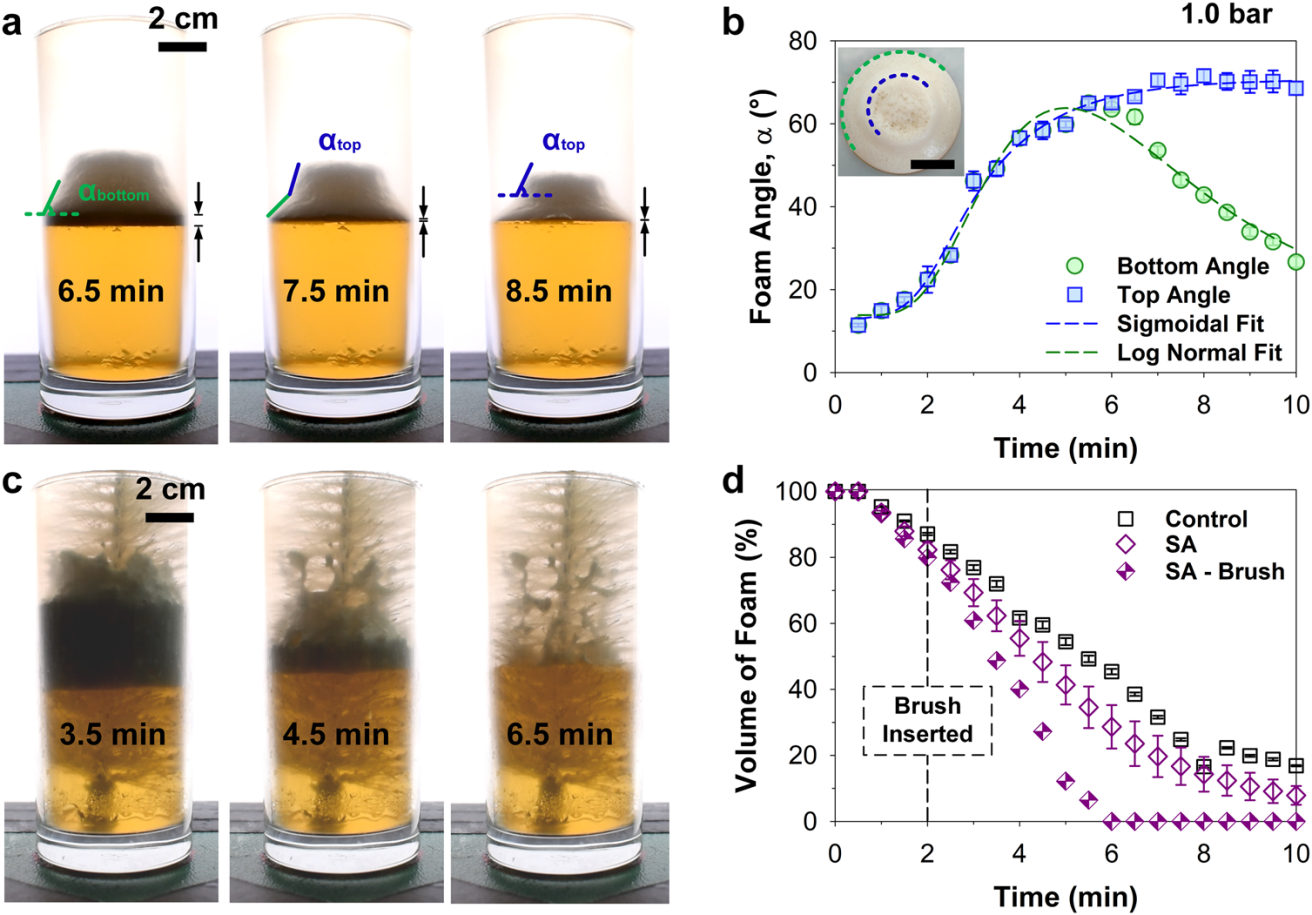
Importance of foam-to-surface contact for defoaming. **a** The foam-to-surface contact line recedes from the superamphiphobic (SA) surface due to rapid destabilization. An angular cone-like structure first evolves, bearing a single angle. **a**, **b** The single angle degrades into two distinctive angles during continued defoaming of the volumetric bulk. Inset—top view, scale bar: 2 cm. **c** A three-dimensional volumetric defoamer was synthesized using a high surface area template, i.e., a brush coated with the superamphiphobic coating. This was compared to the original superamphiphobic and control systems. **c**, **d** The superamphiphobic volumetric defoamer experienced up to 40–50% defoaming enhancement *vs*. the control. Dispensing pressure: 1.0 bar using contrasted backlighting. All data are presented in mean ± standard deviations.

Functionally, the presence of airgaps/-channels facilitates the easy escape of a film-destabilized bubble, enabling cycling of the process. As soon as a bubble has burst, a neighboring bubble moves to the vacated area (Supplementary Movie 6). Bursting of bubbles at the interface results in fast defoaming. However, in the center of the glass, defoaming is still dominated by slower buoyancy-induced processes. The differences in surface and volumetric defoaming rates reshape the foam column. The foam column forms a truncated cone, receded from the superamphiphobic coating (Fig. 5a, shadowgraphs). After the foam column has delaminated from the sidewalls at *ca*. 6.5 min, the slope of the foam column buckles, forming a kink (Fig. 5b) with two distinctive slopes (angles). This occurs due to the finite elasticity of the cone’s solid-like structure, resulting in it collapsing (Fig. 5b, inset) on its own weight. A receded foam-to-surface contact line takes place alongside the formation of the cone-shaped foam column. This results in a shrunken volumetric bulk that does not contact the functional superamphiphobic surface. As observed in Fig. 5a, b, at beyond 6 min, defoaming of the stable wet foam is still dominated by the volumetric bulk, thus questioning the relevance of bubble bursting induced only by the surrounding surface.

To circumvent the reduction of defoaming rates caused by a shrinking volumetric bulk, we introduce a three-dimensional volumetric defoamer. This consists of the use of the superamphiphobic coating on a laboratory brush. The bristles increase the net effective surface area within the bulk. The entire brush was rendered superamphiphobic by spray coating under identical conditions used for the cups. The coating deposited on brush bristles gives rise to a three-dimensional interconnected air layer (Fig. 5c and Supplementary Movie 7). The model mechanical defoamer maintained high foam-to-surface contact during the entire defoaming process, thereby increasing the overall rate of defoaming by up to 40–50% (Fig. 5c, d, half-filled purple diamonds). More interestingly, the presence of the bristles at the foam-to-liquid contact line enabled complete defoaming down to the liquid level. A negligible decrease in defoaming rate was observed throughout the process. The superamphiphobic layer induced bubble bursting while the interconnected air layers ensured rapid removal of escaping CO_2_.

### Antifoaming properties

**Fig. 6:**
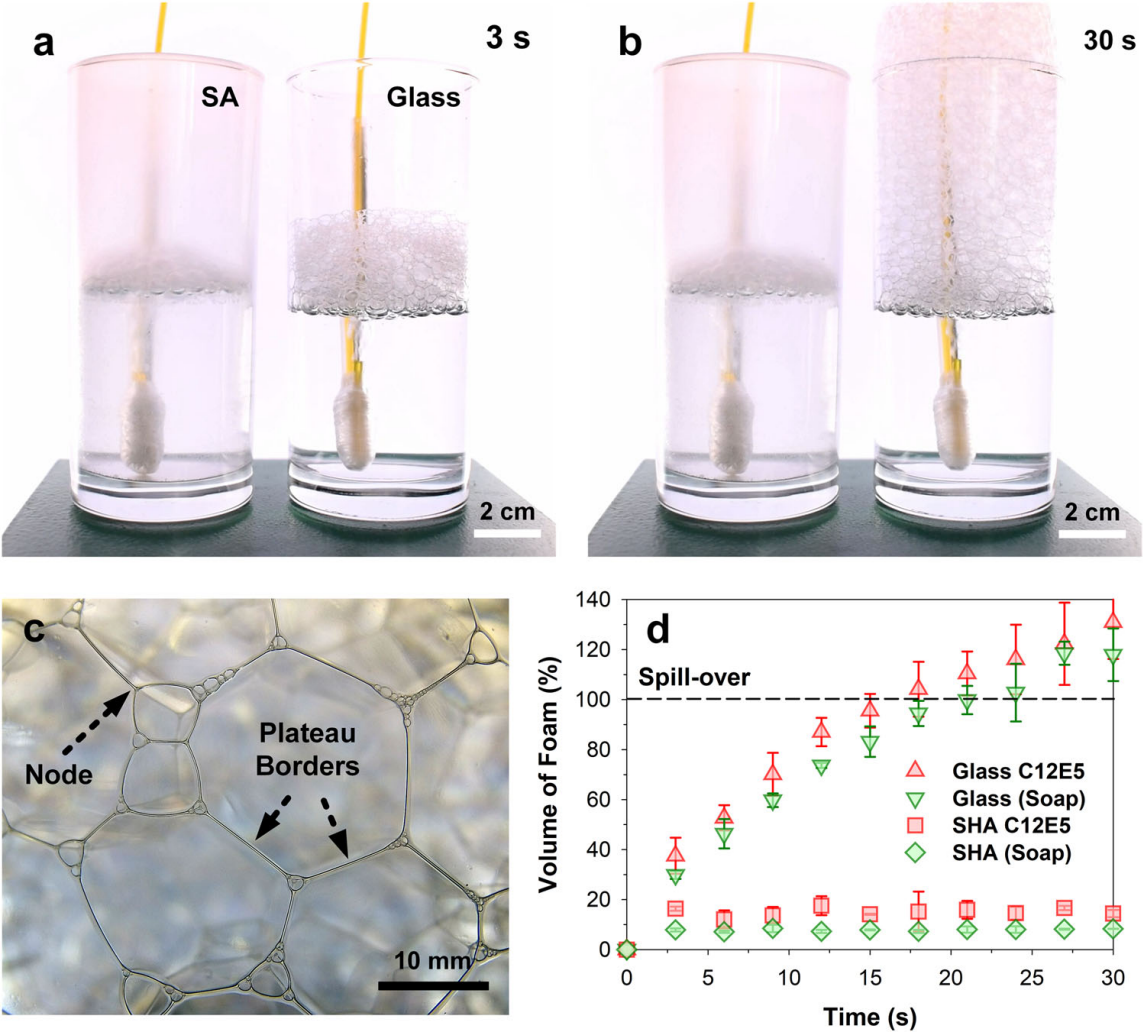
Demonstration of antifoaming and froth control. Antifoaming was quantified using a bubbling setup that delivers *ca*. 5 mL s^−1^ of nitrogen at 0.25 bars through a U-tube at an inner orifice diameter of 0.5 mm. The soap demonstrated here was (**a**, **b**) pentaethylene glycol monododecyl ether (C12E5, critical micelle concentration of 0.03 g L^−1^). The soap was mixed into water at a concentration of 0.4 g L^−1^. **c** Photo of foam bubble close to the surface. The Plateau borders refer to the channels where films meet. The nodes refer to places where four channels meet. The formation of (**d**) dry foams in the superamphiphobic glasses (SA) was suppressed (<10% of total available volume) compared to the control glass (glass). Beyond 30 s, foam in the control system spills over, and further observation was halted. All data are presented in mean ± standard deviations.

To investigate the use of superamphiphobic surfaces for antifoaming (suppression of foam formation), bubbling of soapy water (0.25 bars through a tube) was performed within the superamphiphobic cups. Two soaps were tested, the nonionic surfactant pentaethylene glycol monododecyl ether (C12E5) (Fig. 6a, b and Supplementary Movie 8) and commercial dishwashing soaps (Supplementary Fig. 8 and Supplementary Movie 9), which are a mixture of cationic, anionic, and nonionic surfactants of various molecular weights. Soap foams are composed of dry foam cells (Fig. 6c), unlike the stable liquid foam bubbles observed in beer, Fig. 3d. Soap foams can also be continuously aerated to simulate uncontrollable foam formation and growth.

Superamphiphobic surfaces suppressed the formation of foams above the liquid line. In uncoated glasses, foams grew with an almost linear behavior, resulting in spillover after 30 s (Fig. 6a, b, d). Compared to controls (glass), the superamphiphobic surfaces suppressed foaming up to a measured level of larger than 100%. In fact, superamphiphobicity appears to be capable of limiting the maximum height and volume of a spontaneously foaming column by interfacial destabilization. Dry foams burst almost immediately (at ≪1 s after surface interaction) upon contact with the superamphiphobic surface. This is expected since no liquid drainage (as in wet foams) is necessary before the formation of unstable films (Fig. 6d). This holds for all pressures investigated, i.e., up to 1.0 bar.

In summary, superamphiphobic surfaces are capable of actively defoaming in situ generated wet liquid foams as well as inhibiting dry foam formation. This additive-free and energy-efficient method for defoaming and antifoaming processes can be particularly important for the food and chemical industries. Advantages of superamphiphobic surfaces for defoaming, antifoaming, and froth control are: (a) superamphiphobic surfaces are not affected by repeated use with foaming liquids. (b) They do not require or result in the leakage/release of material into the two-phase foam. (c) They are easily scalable with current methods.

## Methods

### Synthesis of super liquid-repellent glasses

Cylindrical glasses (internal diameter: 5.5 cm, height: 12 cm) were washed in ethanol and dried using a nitrogen gun. When unfunctionalized, the untreated soda-lime glass represents the control.

Glass modified with a liquid-like coating was prepared according to the procedure reported by Wang and McCarthy^22^. These consist of liquid-like PDMS brushes (LL-PDMS) bonded to the glass, which were synthesized using dimethyldimethoxysilane as the precursor. A solution containing sulfuric acid (1.3 mL, 95–97%, Aldrich) and dimethyldimethoxysilane (23 mL, 95%, Aldrich) in isopropanol (300 mL, 99.8%, Fisher) was prepared half an hour before use. The solution was poured into the glass and allowed to stay for 10 s. After this time, the liquid was removed and the borders of the glass were quickly wiped. Immediately after, the glass was placed in a closed chamber for 20 min at a relative humidity of 62%. Before concluding the experiment, the glass was not washed; this means that a layer of unbonded PDMS was still present during subsequent experiments.

Slippery liquid-infused surfaces (SLIPS) were synthesized from a scalable design using commercial *glaco* suspensions. The textured glass slide was gently coated with a solution of Glaco Mirror Coat^®^ (soft 99) applied by rinsing the sample surfaces with the suspension using Pasteur pipettes followed by manually distributing the suspension. It consists of nanobeads (size: 30 nm) suspended in isopropanol. The solvent is evaporated by placing the coated surfaces in a furnace held at a temperature of 70 °C for 30 min. The deposition cycle was repeated three times in order to obtain a homogeneous coating. This procedure leads to the formation of a layer of nanobeads. The slippery liquid-infused porous surfaces (SLIPS) variant comprises of a 2-µm-thick nanoparticle layer (Glaco) as determined by SEM. In total, 5 mL of hexadecane (4.5 cSt, Aldrich), silicone oil (5 cSt and 500 cSt, Gelest, vinyl-terminated polydimethyl siloxane), and sunflower oil (Ja!, Rewe) were spread over the internal surface area of the glass (*ca*. 230 cm^2^) before removal of the excess by resting the glass upside down for *ca*. 5 min. The lubricants and associated surface tensions: silicone oil, 500 cSt and 5 cSt (SO-500cSt, SO-5cSt), ${\gamma }_{{{{{{\rm{PDMS}}}}}}-{{{{{\rm{A}}}}}}}$ = 21 mN m^−1^, hexadecane, 4.5 cSt (HD), ${\gamma }_{{{{{{\rm{HD}}}}}}-{{{{{\rm{A}}}}}}}$ = 27.5 mN m^−1^ and sunflower oil, ${\gamma }_{{{{{{\rm{SFO}}}}}}-{{{{{\rm{A}}}}}}}$ = 35 mN m^−1^ ^21^. The silicone oil variants enables the assessment of the influence of the oil viscosity while hexadecane and sunflower oil were used to investigate the influence of the interfacial tension and chemical compatibility between the lubricant and beer on defoaming.

Superamphiphobic glasses were created by a two-step spray-coating procedure. The first layer is a binder, composed of a simple polymer-in-solvent mixture (5 mg mL^−1^ polystyrene in acetone–toluene (1:1 co-solvents), *M*_w_ = 1,300,000). The second layer is a superamphiphobic powder coating. The superamphiphobic powder is prepared as follows: 1 g of fumed silica (Aldrich, diameter 7 nm) is stirred with 30 mL of chloroform (Aldrich, >99%). In all, 0.81 mL of heptadecafluoro-1,1,2,2-tetrahydrodecyl trichlorosilane (TCI, >96%) is then added to the solution and stirred for 72 h. These steps are performed in a glove box with a nitrogen purged atmosphere. The resulting functionalized nanoparticles, termed superamphiphobic silica, are retrieved, washed three times in 30 mL of chloroform, and dried in a vacuum oven (50 mbar, 60 °C) for 24 h. The superamphiphobic silica is then redispersed in acetone (10 mg mL^−1^) under stirring (1 h) and sonication (30 min) before spray coating.

A total area of 230 cm^2^ needs to be coated per glass, which represents: 23 mL of polystyrene in acetone–toluene (5 mg mL^−1^) with 34.5 mL of superamphiphobic silica suspension (10 mg mL^−1^). Both layers were sprayed onto the surface sequentially at *ca*. 0.2 mL s^−1^, at a pressure of 3 bars, under an approximate working distance of 10 cm. The polystyrene layer is allowed to dry for *ca*. 10 min before deposition of the second superamphiphobic coating. The particles were integrated into the binder via mechanical penetration. The superamphiphobic surfaces did not degrade over time as they remained functional over multiple cycles (tested up to ten defoaming cycles, under immersion of up to 10–15 min per cycle).

### Wetting analysis—contact angles and beer drop analysis

Flat glass substrates were prepared (uncoated controls, superamphiphobicity, liquid-infused, and liquid-like coatings) based on the above steps. Static contact angles are recorded using the sessile drop method (5 µL of Bitburger Pilsner beer, dispensed at 0.5 µL s^−1^, Data Physics OCA35 goniometer). Sliding contact angles are typically <10° for both the liquid-infused (silicone oil) and superamphiphobic surfaces. These tests were performed up to a tilt angle of 10°, with a tilt speed of 1° s^−1^. The contact angles and sliding angles were computed by a commercially available program (SCA). All data were presented as mean ± standard deviations over three measurements. The surface tension of the Bitburger Pilsner beer was measured using the pendant drop technique, using a drop of *ca*. 4 μL (Krüss DSA100). A needle of an outer diameter 0.285 mm was used. The surface tension of between 43 and 44 mN m^−1^ was recorded.

### Foam-dispensing experiments

Commercial beer (Bitburger, Germany, 5 L kegs) was used as a standard foam liquid because of its easy handling and good foaming reproducibility^43,44^. First, the valve from the sealed keg is opened, dispensing a flush volume of 1 L (foam and beer). Thereafter, a hole, coupled to a pressure valve, is drilled into the top cap of the beer tank. This is then re-pressurized using compressed nitrogen gas, at three specific ratings: 0.1, 0.5, and 1.0 bar. Nitrogen serves only as the carrying gas^43^. The nozzle is then adapted to a flexible tubing of fixed length and internal diameter (*ca*. 0.75 cm). A further test run of at least 500 mL is used to ensure smooth and reliable pressurized flow. A new tank of beer is used if a series of experiments last more than 5 h or is down to the last 1 L of beer. In all experiments, the impact height of the beer (from the tip of the fitted nozzle) to the bottom of the glass is fixed at 15 cm. The volumetric flow rates at the three different pressures (1, 0.5, and 0.1 bars) were experimentally measured at 86 ± 1, 74 ± 2 and 24 ± 1 mL s^−1^ respectively. At 0.1, 0.5, and 1.0 bars, the glasses were filled within 11, 5, and 3 s, respectively.

### Foaming and Imaging

A full-view mode was first designed for imaging beer dispensing and foam evolution for the entire dimensions (internal diameter: 5.5 cm, height: 12 cm) of the glass. A dynamic single-lens reflex camera (Nikon D3300) was used in the manual mode: with an aperture of F3.5, shutter speed of 1:100, ISO 400 at 50 frames per second at 1920 × 1080 p resolution. The focus was always adjusted to the central axis of the glass, at *ca*. *X* axis of 2.75 cm and *Z* axis of 6 cm. This was determined based on pre-runs that confirmed the region of foam dissipation. Ten  minutes of recording (maximum setting) was used to capture all images in color based on backlighting (diffused LED panels) assisted shadowgraphy. As most defoaming methods result in some sticky foam residuals, the amount of contaminant residuals in each dominant system was also imaged and presented (Supplementary Fig. 6).

A macromode was then later employed using home-built glass cups, fabricated at dimensions of 2.5  × 2.5  × 7.5 cm using optical glass. The focus was fixed at 5 cm above the bottoms of the cups, and 2.5 cm below the tops, or at the 75% height of the system. A dispensing height of 15 cm at 1.0 bar was employed to ensure consistency between measurements. Backlighting was supplied via a SCHOTT KL2500 LCD at maximum brightness. Videos were captured on a home-built portable board camera (SONY IMX 179, Allwinner V3) with manually adjustable focus using a M12 lens. Focus was always pre-set before the actual experiment by a trial run, enabling an adjustment to the solid-bubble interface. A video resolution of 2440 × 2440 p (2.2 mm per 1080 pixels) was captured, at 30 frames per second. Due to aberrations, only a crop of the central area (3.5 mm^2^) was used for analysis. A scale bar was determined by the imaging resolution. Four minutes of recording was used to capture the entire progress of the receding foam line, past the 75% height limit for all samples.

### Computational analysis—beer and foam volumes

A MATLAB script was written that combines the computation of foam and beer dimensions with respect to time from the full-view imaging of the glasses during dispensing (Supplementary Fig. S3). The first script allows the determination of foam heights (regular/irregular) based on the foam’s wetting progression line. The 2D plane or heights obtained are then circumferentially rotated to form a cylindrical 3D profile, enabling the computation of dynamic foam volumes. The second script allows independent determination of beer heights, which typically exhibits a regular progression upward as a liquid contact line. The 2D plane, or heights obtained are then circumferentially rotated to form a cylindrical 3D profile, enabling the computation of dynamic beer volumes. Scale bars are determined based on the in-image scale, captured in the images. *t* = 0 s measurements are assumed to be 100% since dispensing takes between 3 and 12 s, while the frame captured at 30 s was used to normalize all subsequent frames. MATLAB thresholding packages such as graythresh and adaptive thresholding are used, in combination with the segmentation image processing toolbox.

### Computational analysis—bubble population and dimensions

A second script was written to evaluate the behavior of foam in the macromode. This code aims to recognize every bubble in every frame of the video, enabling a precise computation of foam evolution under different conditions (Supplementary Fig. 9). First, the video was prepared with Fiji using special filters (Supplementary Movie 10) so that every recognized bubble is outlined by a connected one-pixel thick line. Then, outline pixels containing three or more neighbors are deleted. This step filters very close bubbles that form a connected pixel structure. This filter is not used for large bubbles, particularly for liquid-infused surfaces. Thereafter, the connected pixel structure is fitted to a circle. When the number of pixels do not exceed a quarter of the as-calculated circle perimeter, this circle is omitted. This facilitates noise detection and elimination. When two or more circles are found to be concentric, circles are merged. If one circle is clearly centralized, the other circle is omitted. This prevents the interruption of bubble outlines. Sudden changes can be detected and aligned to either coarsening or bursting events. These were manually recorded and evaluated frame-by-frame until the foam line has receded out of view. The starting point of the evaluation was taken at 12 s on all systems due to the liquid swirling dynamics immediately after foam deposition. Notably, the low adhesion properties of the superamphiphobic glass resulted in a longer dynamical motion (slip) during liquid deposition (Supplementary Movie 6).

### Antifoaming under dynamic dry foam growth

Bubbling liquids were composed of soaps dispersed in water. The first variant was the nonionic surfactant pentaethylene glycol monododecyl ether (C12E5), while the second variant was a commercial soap (Greencare, Manudish Original). Both soaps were mixed into water at a ratio of 0.4 g L^−1^. A bubbling setup was constructed using a U-shape tubing, having an internal diameter of 0.5 mm, suspended at the bottom of the chamber. Nitrogen is purged through the tubing at a pressure of 0.25 bars. This creates a wave of bubbles that floods the respective chambers. Foaming/defoaming behaviors were then dynamically observed and monitored by optical videography.

### High-speed optical imaging of single-bubble rupture dynamics

The approach and bursting of single bubbles encountering a superamphiphobic surface were analyzed optically using a high-speed camera (20,000 fps, Photron Fastcam Mini AX10, Japan). The coated surface was immersed (inverted) in (1) deionized water, (2) ethanol/water mixture (5/95 v/v ratio), and (3) beer (original foam liquid). The coated side of the superamphiphobic surface was facing down. We released air bubbles with the help of an underwater syringe at varying depths (0.28–73.7 mm). The approach velocity was tuned by the distance between the release position and the surface. The rupture time refers to the time between contact (determined by the local minimum of the velocity–time graph) of the bubble with the surface and its rupturing (determined by the local maximum of the velocity–time graph).

### Holographic imaging of single-bubble rupture dynamics

In this experiment, immobilized bubbles were moved toward a superamphiphobic surface (at 5 µm s^−1^ approach velocity) using a micromanipulator. The thickness of the thin liquid film between the bubble and the surface was recorded optically by digital holography microscopy (Transmission DHM, T-1000, Lyncee-tec, Switzerland). Different refractive indices of the liquid and gas phases increase the optical path of an illumination beam (wavelength: 666 nm). This increase is apparent when compared with a reference beam. A CMOS sensor captures the optical transmission signal of phase difference between the illumination and reference beam every 5.5 ms. The phase difference allows for the extraction of changes in the thickness of the thin film within nanometric resolution. We reconstructed the heights in the field of observation by unwrapping the phase difference, using the scikit-image processing library in python. Similarly, the heights over time were obtained by unwrapping the temporal changes of phase difference. We recorded film thinning, including the point of rupture at the end of the thinning process. For beer, a steady plateau in film heights was noted before a sudden change in height. This was followed by two frames (11 ms) in which the illumination path only travels through the superamphiphobic surface and the air within the ruptured bubble. Thereafter, the phase difference signal loses coherence, which is caused by the flooding of liquid. We determine the absolute film height before rupture by referencing the phase difference signal before rupture (with liquid film) to the subsequent frame (no liquid film).

### Determination of noise and film height uncertainties

During the experiment, various environmental perturbations interfered with the experiment, giving rise to noise. We determine three main sources: (1) External vibrations: the two frames after film rupture (11 ms) should, in reality show the same film configuration. However, external vibrations from the surroundings reveal an error in travel distance (*ca*. 80 nm). (2) Time lag: the film rupture occurs between two recorded frames (5.5 ms). The film might have thus traveled further towards the superamphiphobic surface before spontaneous rupture by hydrophobic interactions. Considering the frame rate of 5.5 ms and prior thin-film travel velocity (from previous frames), the maximum uncertainty in travel distance is *ca*. 20 nm. (3) Shot noise: shot noise was determined by averaging the near-zero values measured within the total height distribution of each frame. On average, this value was 3.3 nm. Assuming independent variables, the total error propagation is, $\epsilon =\sqrt{{\left(80\,{{{{{\rm{nm}}}}}}\right)}^{2}+{\left(20\,{{{{{\rm{nm}}}}}}\right)}^{2}+{\left(3.3\,{{{{{\rm{nm}}}}}}\right)}^{2}}=83\,{{{{{\rm{nm}}}}}}$.

### Interference measurements of liquid–solid contact lines

Due to the reduced surface tension, beer can be expected to penetrate deeper into the coating as compared to pure water. To measure this excess depth, Δ*h* (Supplementary Fig. 7), we set up reflection interference microscopy (RICM) on an inverted confocal microscope (Leica TCS SP8, Wetzlar, Germany) using ${\lambda }$ = 561 nm and 633 nm lasers with a low numerical aperture objective (Leica HC PL APO 10×/0.4). A coating of ~5 µm thickness was deposited on a 170-µm coverslip. A 30 µL drop of water was first deposited on a superamphiphobic surface. Light reflected from the coverslip/coating interface and the coating/water interface interfered, generating reflected light images (Supplementary Fig. 7a–c). The liquid contact line within the measurement time (100 s) was stable. Interference patterns show that the contact line (water-to-surface) is inclined. The field of view was *ca*. 100 × 100 µm. The focus was set at 5 µm above the surface. Time resolution between scans was fixed at 227 ms. Using a syringe pump, we slowly injected 6 µL of ethanol into the 30 µL water drop (0.146 µL s^−1^). This changes the liquid surface tension from (72 mN m^−1^ down to 44 mN m^−1^, as an analogy to beer). In our control, we also injected 6 µL of water (Supplementary Fig. 7a–c vs. S7d–f). We observed a continuous shift of the interference stripes upon the addition of ethanol, an effect that was not present in our control experiment. We interpret the shift as the penetration of liquid into the coating. A shift of the interference fringes from one maximum to the next corresponds to a progressive penetration into the coating by ${\lambda }$/2. For this calculation, an effective refractive index of 1 was assumed. Therefore, results may represent an underestimate (porous fluorinated material) by <10%. The injection takes place for *ca*. 25–30 s. A time lag of a few seconds between the starting of the pump and actual injection occurs due to pressure build-up in the tube. We evaluated the shift of the interference fringes in ImageJ, drawing lines perpendicular to the interference fringes as shown in Supplementary Fig. 7. The intensity profiles along these lines are captured from video frames. A 3D intensity graph, from which the continuous shift ${\Delta }x$ of fringes can thus be evaluated (see red guidelines in Supplementary Fig. 7c, f). The final height difference, Δ*h* from these geometrical considerations are evaluated as, ${\Delta }h=\frac{{\lambda }{\Delta }x}{2{\Delta }F}$ with ${\Delta }F$ being the distance of two adjacent intensity maxima. This shift was analyzed over multiple data sets (two sets × five line fringe scans) for both water (control) and ethanol (beer analog). This shift revealed that the liquid contact line (thus, film) moves into the surface by an average wetting depth of down to 350 ± 220 nm (Supplementary Fig. 7).

### Light-scattering measurements

Light-scattering measurements were performed on an ALV spectrometer consisting of a goniometer and an ALV-5004 multiple-tau full-digital correlator (320 channels) which allows measurements over an angular range from 30 to 150°. A He-Ne Laser (wavelength of 632.8 nm) is used as the light source. For temperature-controlled measurements, the light-scattering instrument is equipped with a thermostat from Julabo. Diluted dispersions were filtered through low protein binding hydrophilic PTFE membrane filters with a pore size of 0.20 µm (LG Millipore). Measurements were performed at 20 °C at 7 (dynamic) angles ranging from 30 to 150°. The ALV/LSE-5004 correlator and ALV5000 software were used for data analysis.

## Supplementary information

Supplementary Information

Description of Additional Supplementary Files

Supplementary Movie 1

Supplementary Movie 2

Supplementary Movie 3

Supplementary Movie 4

Supplementary Movie 5

Supplementary Movie 6

Supplementary Movie 7

Supplementary Movie 8

Supplementary Movie 9

Supplementary Movie 10

## Data Availability

All data generated or analyzed during this study are included in this published article and its Supplementary Information files, including Supplementary Movies. Source data are provided with this paper.

## Source data

Source Data

## Code availability

A MATLAB script and sample pictures for foam height analysis are included in the Supplementary Information files (as a.zip folder).
