# Supplementary material for: Super liquid repellent surfaces for anti-foaming and froth management
Source: Nat Commun. 2021 Sep 9;12:5358. doi: 10.1038/s41467-021-25556-w (PMC8429590; doi:10.1038/s41467-021-25556-w)
Supplement: Supplementary file 1 — Supplementary Information [file 41467_2021_25556_MOESM1_ESM.pdf]

Supplementary Information for

Super Liquid Repellent Surfaces for Anti-Foaming and Froth Management

William S. Y. Wong, Abhinav Naga, Lukas Hauer, Philipp Baumli, Hoimar Bauer, Katharina I. Hegner, Maria D'Acunzi, Anke Kaltbeitzel, Hans-Jürgen Butt and Doris Vollmer**

Max Planck Institute for Polymer Research, Ackermannweg 10, D-55128, Mainz, Germany

Keywords: Lubricant infused surfaces, Slippery surfaces SLIPS, Superamphiphobic surfaces, De-foaming, Bubble destabilization.

This file includes:

Supplementary Figures 1 to 11
Supplementary Discussion
Supplementary References

Supplementary Figures

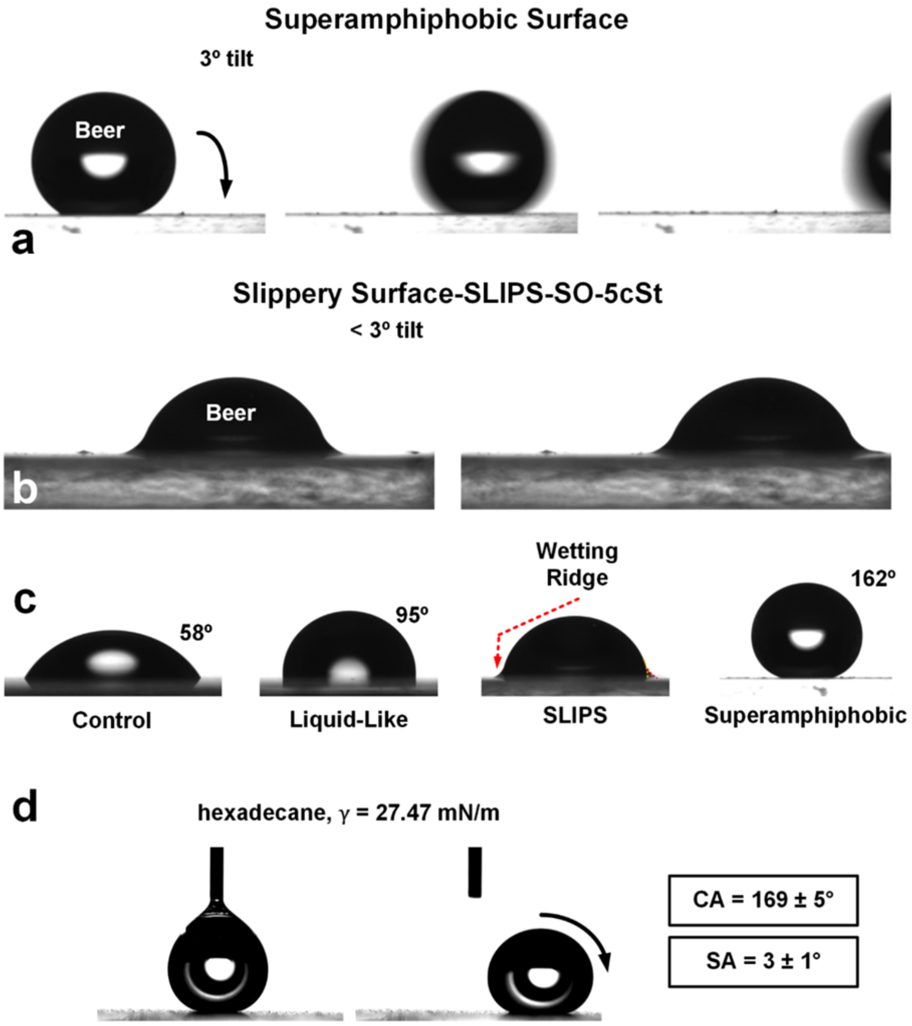

Supplementary Figure 1. Liquid repellent behaviour of a drop of beer on the a) superamphiphobic surface (SA) and the b) slippery surface (SLIPS-SO-5cSt). c) The apparent static wetting contact angle of a 6 μL beer drop on these surfaces range from approximately 58° (hydrophilic) to 162° (superamphiphobic). The wetting ridge prevents measuring the contact angle of beer on a slippery surface[1]. d) Contact and sliding angle properties of superamphiphobic surface with hexadecane. SO refers to silicone oil.

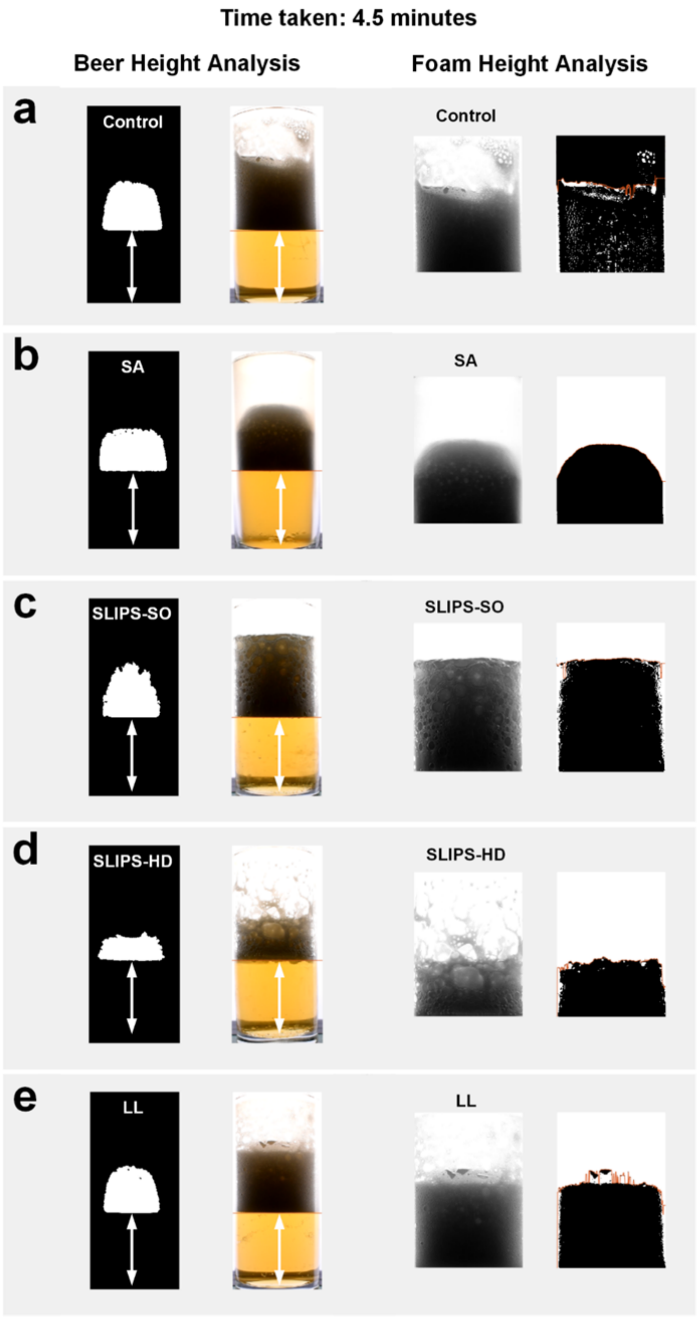

Supplementary Figure 2. MATLAB automated scripts for computing the foam heights (right, XZ axes) and beer heights (left, XZ axes), indicated by the machine-output of a brown colored line in measured height(s). White arrow lines are superimposed on the left images for reference. Beer heights are uniform while foam heights were non-uniform. Surfaces that are tested are listed in the sequence: a) control, b) superamphiphobic (SA), c) SLIPs-Silicone Oil-500cSt, d) SLIPs-hexadecane-5cSt, and e) thin 3 nm liquid like (LL) PDMS-brush like surface. Images are taken using backlighting. SO refers to silicone oil.

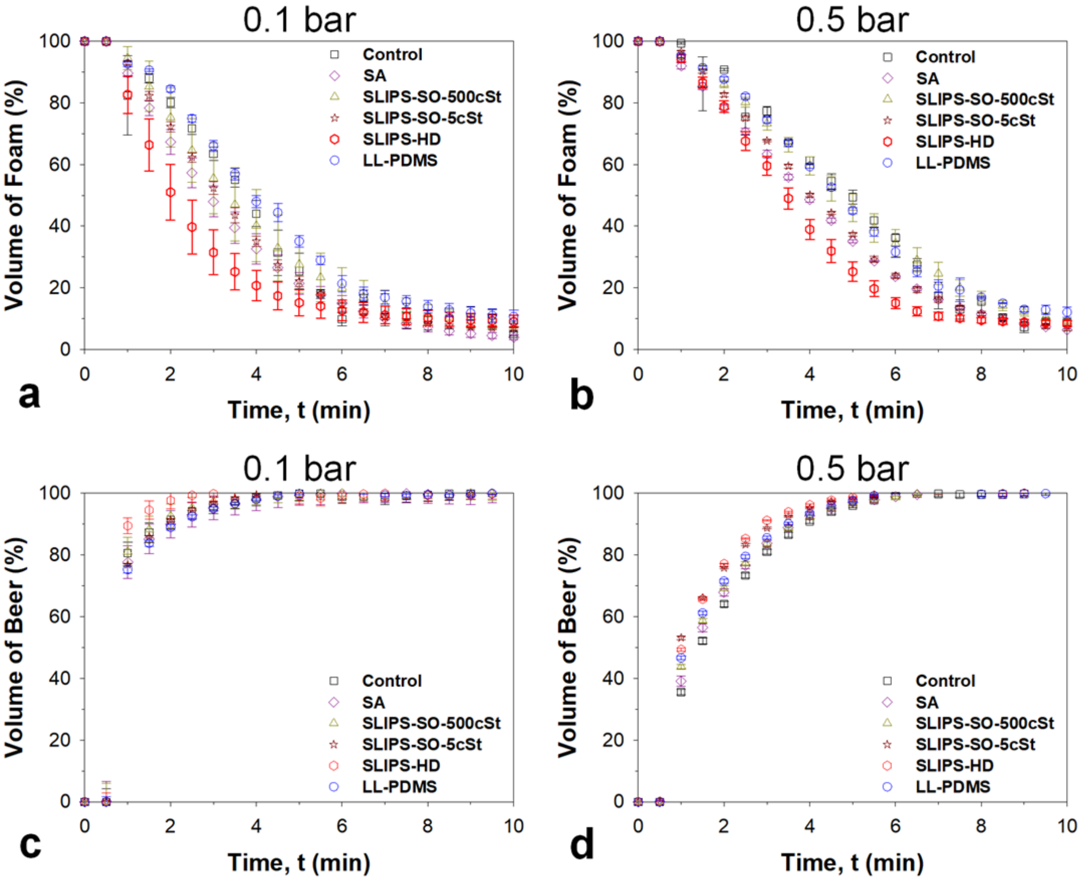

Supplementary Figure 3. Supplementary analysis of a-b) foam and c-d) beer volumes dispensed at a-c) 0.1 bar and b-d) 0.5 bar respectively. The trend of defoaming at various dispensing pressures closely mirrors that of the dispensing pressure a-b) at 1 bar, with the most exemplary performing system as Slippery Liquid Infused Porous Surfaces (SLIPS)-HD (red hexagons), closely followed by superamphiphobic surfaces (SA) (purple diamonds). Liquid-like surfaces (LL-PDMS) performed notably poorly as compared to 1.0 bar. c-d) The formation of beer volume reaches the maximum quickly in these low-pressure dispensing systems due to lower foam stability. However, they notably suffer from greater variance in dispensing consistency. HD refers to hexadecane, SO refers to silicone oil. All data is presented in mean ± standard deviations.

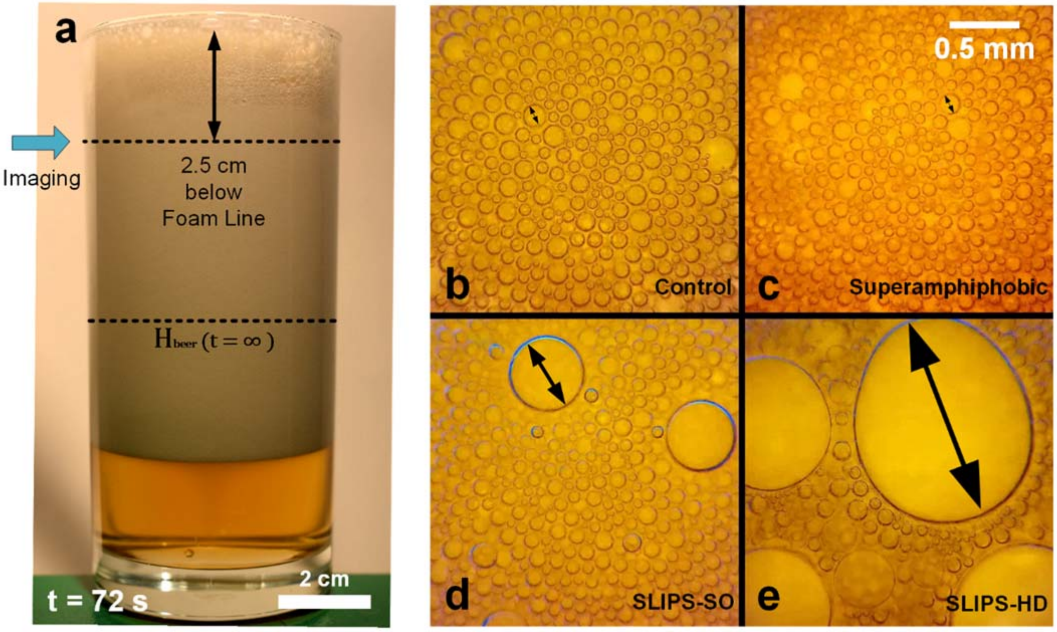

Supplementary Figure 4. Macro-imaging (3.5 mm^2) of bubbles after 72 s of deposition at a) 2.5 cm below the foam-line at the interface, highlighting b) stable foam bubbles in the control, c) small and spontaneously bursting bubbles with a superamphiphobic surface and d-e) coalescence-induced flotation of large bubbles in Slippery Liquid Infused Porous Surfaces (SLIPS). Video-graphical images were taken at this location to document the coalescence and bursting events and bubble geometries. The high liquid content shows that we have a wet foam throughout the entire timespan of observation (Supplementary Movie S6). HD refers to hexadecane, SO refers to silicone oil.

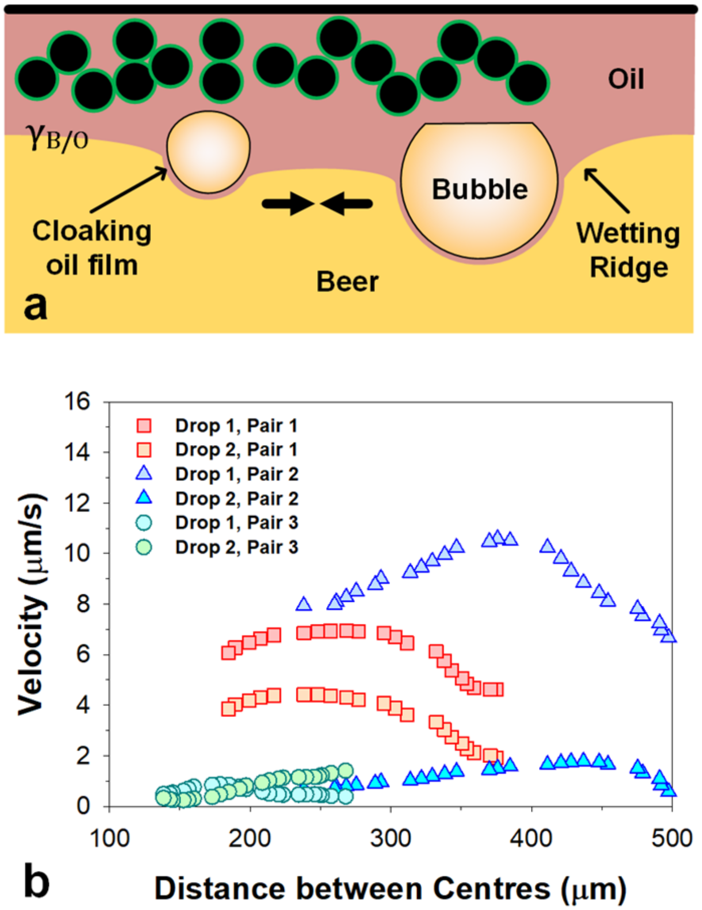

Supplementary Figure 5. Two bubbles in contact with a liquid-infused surface. a) The bubbles are surrounded by a wetting ridge and cloaked in an oil film. $\gamma_{B/O}$ is the interfacial tension of the oil-beer interface. As soon as the wetting ridges overlap, the bubbles attract each other. b) Interaction of bubbles with a SLIPS-SO surface: Long-range interaction of neighboring bubbles on silicone oil infused slippery surfaces, viscosity: 500 cSt. A much higher viscosity was used in order to capture bubble dynamics. Equal size bubbles (Red, Pair 1) and large vs. small bubbles (Blue, Pair 2). Multiple bubble interactions (Green, Pair 3).

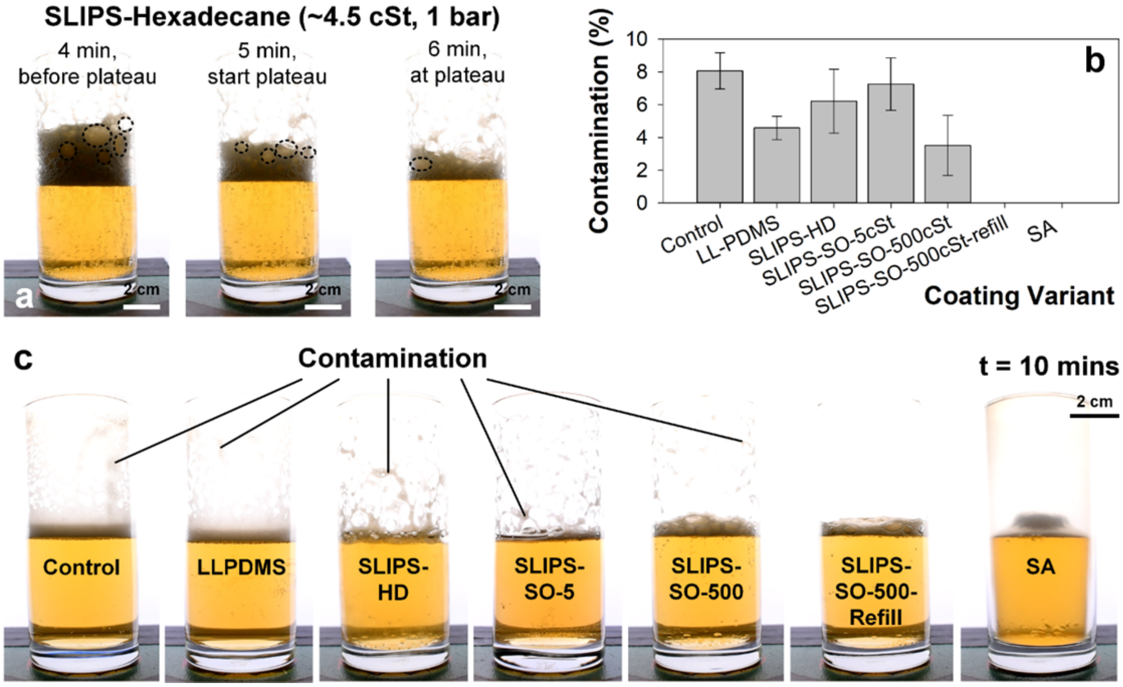

Supplementary Figure 6. Foam-surface interactions under the use of various slippery liquid infused porous surfaces (SLIPS) and liquid-like surfaces (LLPDMS). The behavior of the foam column depletes the liquid-infused surfaces and thus degrades self-cleaning and liquid-repellent properties conferred by SLIPs. a) A layer composed of low viscosity lubricant, such as hexadecane, is particularly affected. Although rapid decomposition of the foam column occurs through direct mixing of hexadecane into the 2-phase foam domain, lubricant depletion results in sporadic losses in coating functionality. In general, the use of low-moderate viscosity oils still suffers from lubricant depletion, owing to the hydrodynamic drag that removes lubricant during flow. As a result, the surfaces are b) not clean from foam after wetting and leaves behind various degrees of c) foam contamination on the side walls (first 5). This occurs in contrast to the c) clean surfaces (last 2) derived from the use of 500 cSt silicone oil that is refilled between runs, or simply: superamphiphobic surfaces. HD refers to hexadecane, SO refers to silicone oil. All data is presented in mean ± standard deviations.

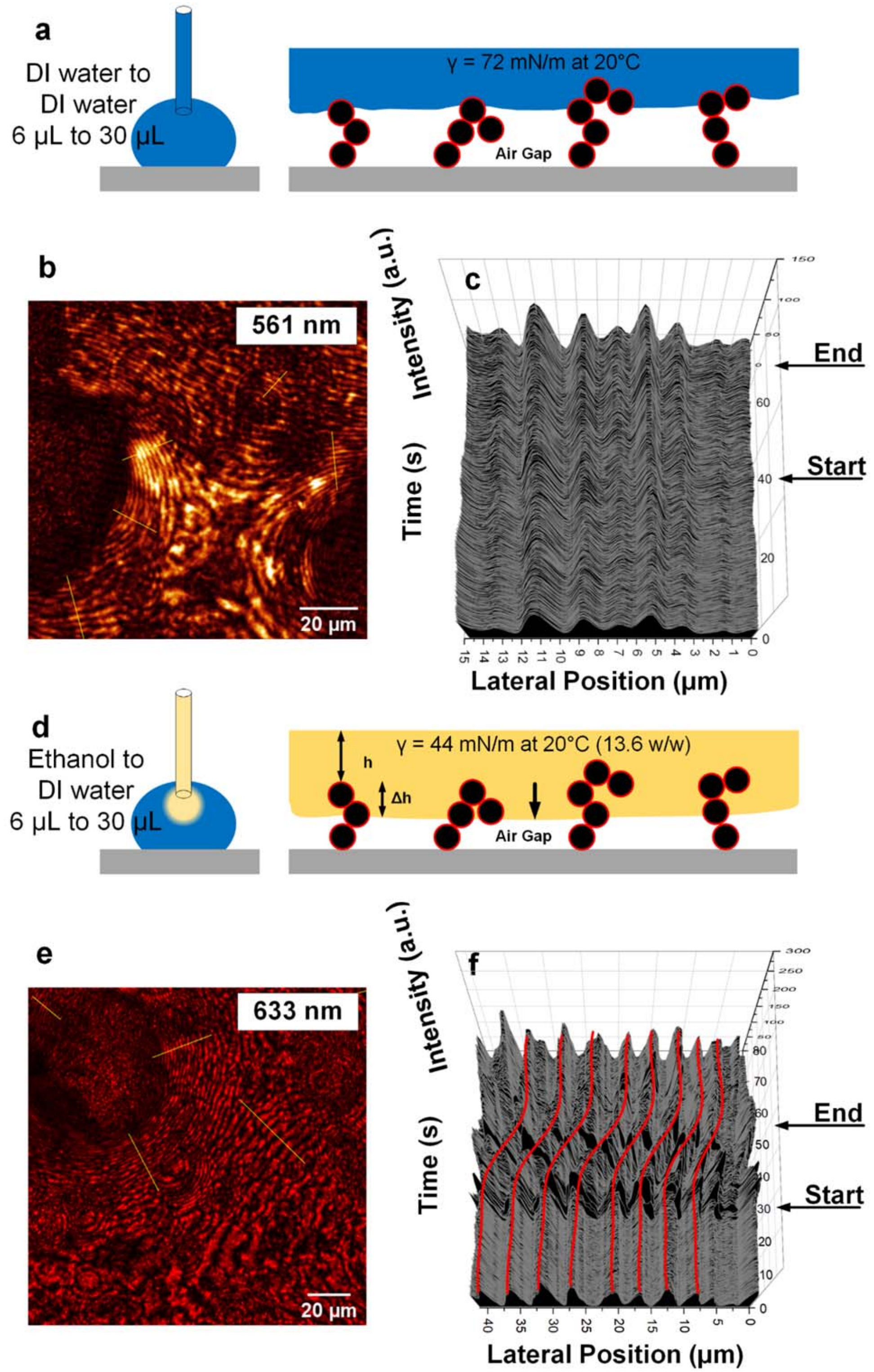

Supplementary Figure 7. Changes in local contact line with an equivalent beer analog (ethanol-water). Interference analysis while adding liquid volumes (6 µL) to a liquid drop (30µL) and dynamically analyzing contact line on protrusions. a-c) Deionized water (DI water) to DI water. d-f) Ethanol to DI water. The shift in interference fringes is interpreted as a motion of the contact line into the porous surface.

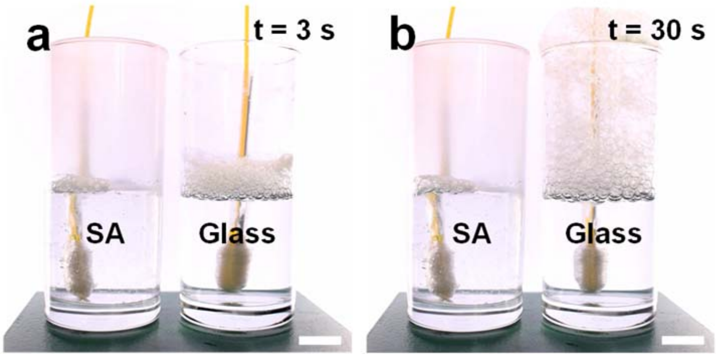

Supplementary Figure 8. Anti-foaming behavior vs. commercial soap foams. The potential of anti-foaming (suppressing foam formation) was also performed using a bubbling set-up that delivers approximately 5 $mL.s^{-1}$ of nitrogen at 0.25 bars through a U-tube at an inner orifice diameter of 0.5 mm. Commercial dishwashing soap, Greencare, Manudish Original was used. The soap was mixed into water at a ratio of 0.4 $g.L^{-1}$. Evidently, the a) formation of foams in the superamphiphobic glasses (SA) were highly suppressed compared to the control glass (Glass). b) Beyond 30 s, foams in the control system spills over and further observation was halted. Foam volumes in the SA glass did not exceed 10% of the total available volume. Scale bar – 2cm.

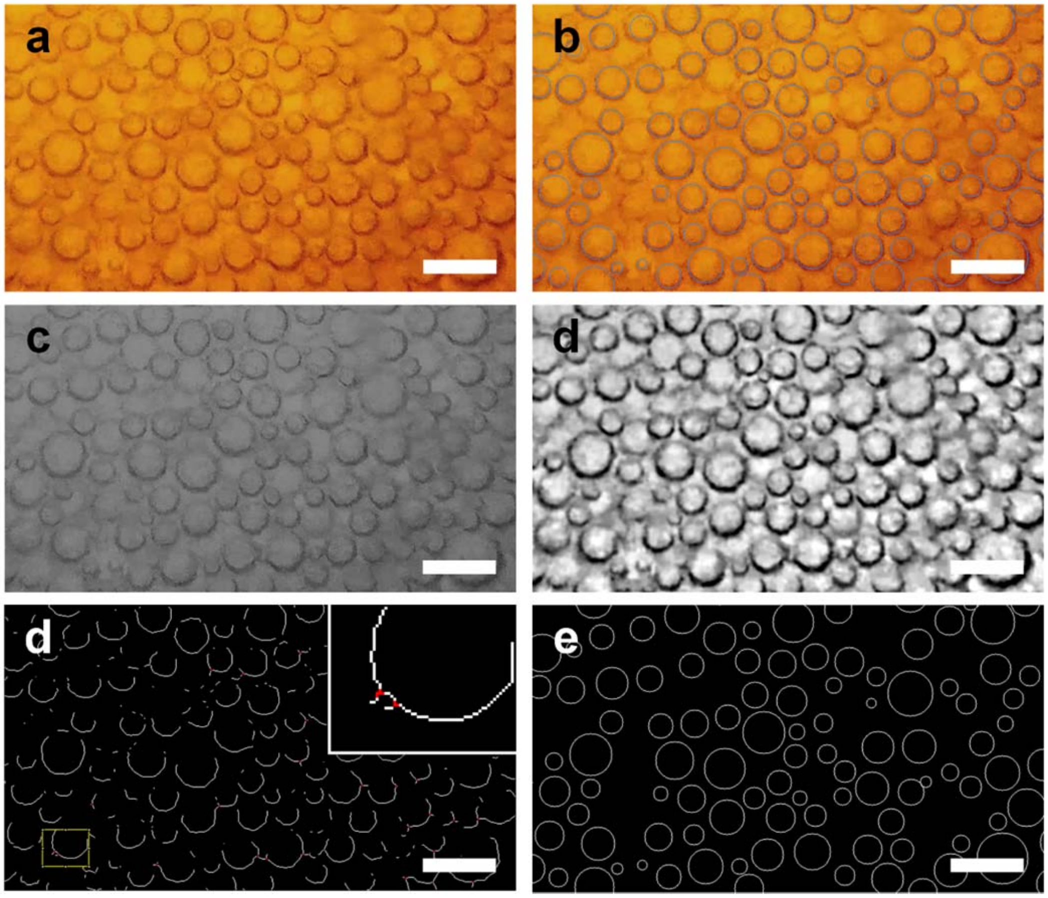

Supplementary Figure 9. Bubble-tracking algorithm. a) Raw image. b) Circles-tracked and fitted. c) Gray-scale image and d) Edge-algorithm. e) Tracked edges of edge-enhanced circles and f) Re-filled circles. Scale-bar - 100 μm.

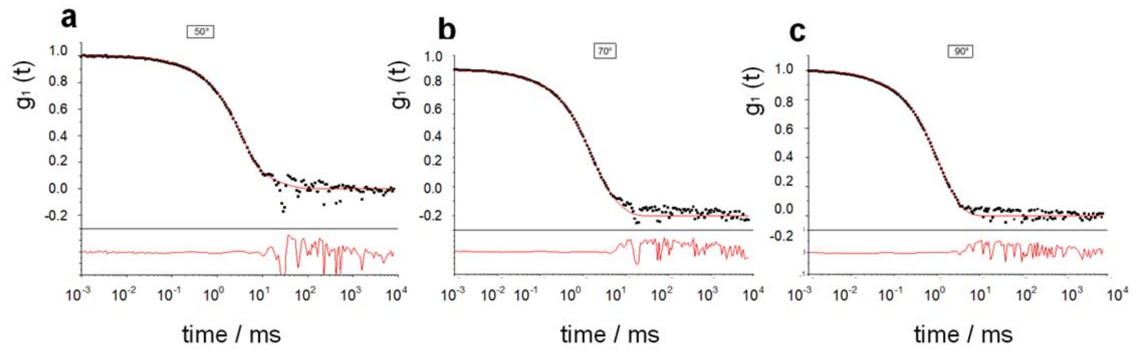

d Intensity Distribution of Multi-component System in Pilsner Beer

Angle	I%1	I% 2	I% 3	Rh 1/ nm	Rh2/ nm	Rh3 / nm
30	2,0	21,6	76,4	1,0	34,9	191,9
50	4,9	66,9	28,2	3,2	77,1	236,1
70	5,4	58,3	36,4	2,2	59,2	212,4
90	5,7	47,8	46,6	2,2	51,6	153,8
110	5,7	18,3	76,0	1,8	27,5	103,0
130	7,1	49,6	43,3	2,1	48,0	145,4
150	7,2	32,2	60,6	2,0	38,3	116,2

Supplementary Figure 10. Size distribution of proteins and particle components in Pilsner Beer. a-c) Correlation function at a) 50°, b) 70° and c) 90°. d) The summarized intensity distribution of multi-component systems in Pilsner Beer[2]. The data reveal that beer contains molecules and aggregates varying between *ca.* 1 nm and 200 nm. Likely, even larger aggregates exist.

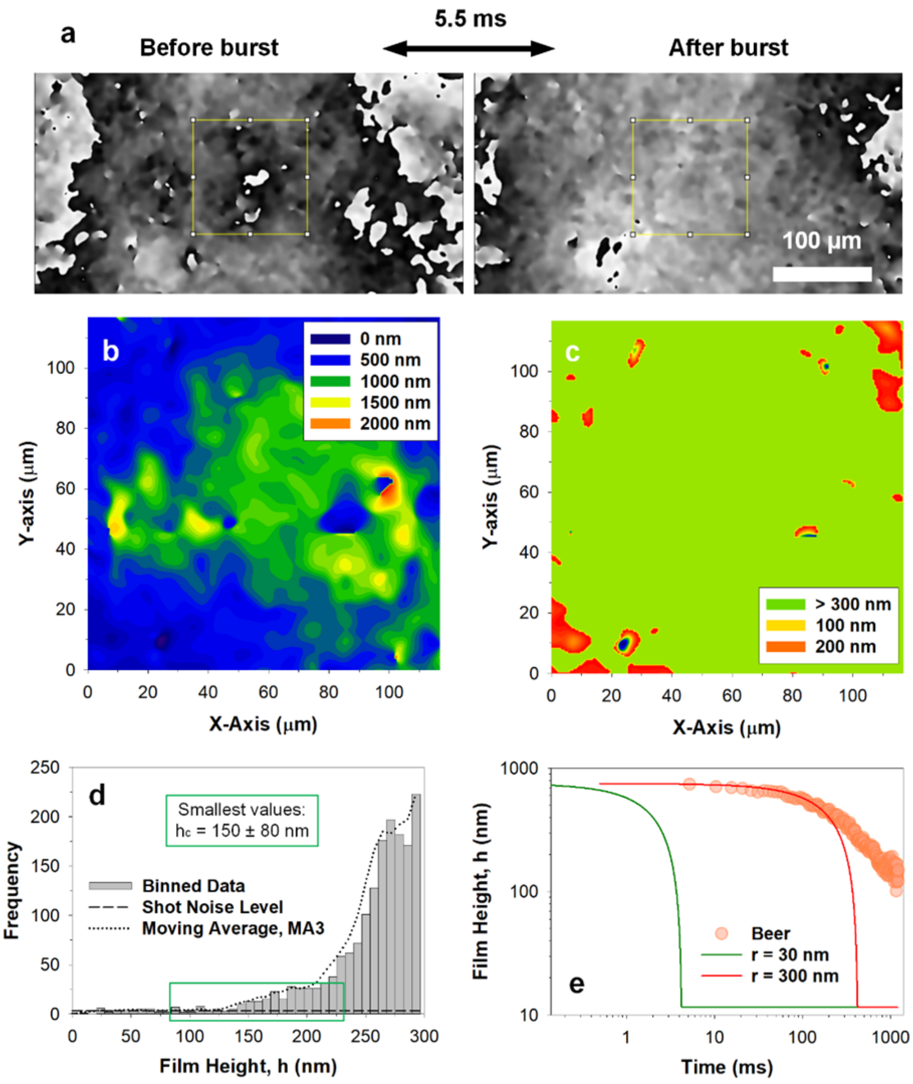

Supplementary Figure 11. Analysis of film heights around agglomerated protrusions in holographic microscopy between a) imaged film rupture. The film around a) agglomerated protrusions (yellow square) can b) vary in heights. The film around a) agglomerated protrusions (yellow square) can b) vary in heights. The c), the smallest critical film thicknesses measured were *ca.* 150 ± 80 nm. d) The binned data comprising of the histogram, the shot noise level (measurement noise) and a moving average (3-points, MA3) are included for reference. e) Attempted modelling of experiment under the Stokes-Reynolds model using a flat film approximation. For the calculation of pressure difference, attractive van der Waals and repulsive electrostatic interactions were considered. The initial condition was selected at 600 nm. At this height, the shape of the film thickness deviates from a linear profile as defined by a constant approach velocity (5 $\mu m.s^{-1}$, Figure 4b, orange circles). Predicted film thinning behavior as solid lines under different protrusion geometry (r = 30 nm, green line and r = 300 nm, red line: as boundary limits). While the model shows the influence of attractive vdW forces, they are not able to predict the precise dynamic behaviors. 1) Model fails to accurately predict the slow-down in film thinning. 2) Model predicts an equilibrium stability at *ca.* 10 nm, but hydrophobic interactions are likely to have induced film rupture at an order before (*ca.* 150 ± 80 nm).[3] The current inability to model both steric repulsion contributions and hydrophobic interactions are likely causing the mismatch between the calculated and measured evolution of the film height.

Supplementary Discussion

1. Superamphiphobicity and Thin Film Interactions

Superamphiphobicity is a surface dominated phenomena.[4] Only the outer-most tips of the nanoparticle coating is in direct contact with the foam films. Immobilized amphiphobic particles exhibit a local contact angles with thin films of approximately at least 90°. High inherent particle-to-liquid contact angles are preferred, estimated to be approximately 80-90° with a regular geometry, but even lesser is required for irregular particles.[5-7]

2. Curvatures and Pressures in the Wetting Ridge

The bubbles in contact with an oil infused wall are surrounded by a wetting ridge.[1,8,9] The pressure in the wetting ridge is dominated by Laplace pressure,

$$P = \gamma_{\mathrm{B/A}} * \left(\frac{1}{r_1} + \frac{1}{r_2}\right) \quad (1)$$

$\gamma_{\mathrm{B/A}}$ is the interfacial tension between beer and air, r_1 is the radius of curvature of the wetting ridge (concave curvature) and r_2 the perpendicular radius (convex curvature). r_1 is smaller than r_2 and negative, resulting in a negative pressure. For the silicone oil meniscus around the water drop resting on the glass surface, the concave curvature along the vertical axis and the perpendicular convex curvature at ~10 µm height above the substrate correspond to radii of $r_1 \approx -230$ µm and $r_2 \approx 1500$ µm, respectively.

Within this manuscript, we define a concave curvature/radius as negative and a convex curvature/radius as positive. From the Young–Laplace equation we get the pressure drop over the meniscus, *i.e.* the Laplace pressure $\Delta P \approx -78$ Pa. However, the pressure changes with height. As soon as the wetting ridge of the neighboring drops overlap, they experience a long-range attraction, Supplementary Figure 5.[10]

3. Spreading and Entry Coefficients

Besides wetting ridges that surround the bubbles, oils are likely to form cloaks around the bubbles that are in contact with the wall. This is because the spreading coefficient is positive (Supplementary Equation 2).

$$S = \gamma_{\mathrm{B}} - \gamma_{\mathrm{B/O}} - \gamma_{\mathrm{O}} > 0 \qquad (2)$$

where $\gamma_{\mathrm{B}}, \gamma_{\mathrm{O}}$ and $\gamma_{\mathrm{B/O}}$ are the macroscopic surface tensions of the aqueous beer phase, oil phase and the interfacial tension of the oil-beer interface respectively. After capillary forces bring bubbles sufficiently close together, low surface tension oil droplets may enter the air-beer interface (bubble film) if the oil droplet's entry coefficient (E) is positive.

$$E = \gamma_{\mathrm{B}} + \gamma_{\mathrm{B/O}} - \gamma_{\mathrm{O}} > 0 \qquad (3)$$

Neighbouring foam bubbles merge if the oil separating both bubbles thins until it ruptures[11,12].[9, 10] Alternatively, spreading of an oil lens[10] on the thin film also induces capillary waves, resulting in locally thinned regions that may rupture easily. For the investigated liquids, γ_{B} = 43.5 ± 0.5 mN.m^{-1}, $\gamma_{\mathrm{B/O}}$ = 15 mN.m^{-1} (HD) and $\gamma_{\mathrm{B/O}}$= 20 mN.m^{-1} (SO). For S > 0, low surface tension oils are needed, γ_{O} < 27.5 (HD) or < 23 (SO) mN.m^{-1}. The coefficients of E are = 31 and 45; and S = 1 and 2 are estimated respectively for hexadecane and silicone oil.

4. Thin Film Behavior described by Stokes-Reynolds Equation

To estimate the time taken for the liquid film between a bubble and a protrusion to rupture, we consider the case of a bubble film pressing against a protrusion. The dominant length-scale is represented by r, which in this case represents the dimension of protrusions. The diameter of the fumed silica used was *ca.* 10 nm, from manufacturer specifications (Sigma Aldrich), but particles can agglomerate during the fabrication of coatings, resulting in measured dimensions of *ca.* 30 ± 10 nm. Film thinning behavior can be described using the Stokes-Reynolds equation.[16-19] This governing equation is based on 2 primary assumptions: 1) low

Reynolds number,[20,21] and 2) no-slip boundary condition (often occurs due to rapid environmental contamination or protein layers in this instance).[14,22] The tangentially immobile (no-slip) boundary condition may be justified by considering the presence of proteins and surfactants in the liquid. Proteins are known to pack along the liquid-air interface, ranging from mono-layers to multi-layers with pH-dependence.[22] Therefore, this makes the contrary (slip) highly unlikely. However, as discussed in the main manuscript, these assumptions are plausible for one specific scenario, but remains yet unproven, and solely serve the purpose to qualitatively present an explicit expression of the timescales observed.

$$\frac{\partial h}{\partial t} = \frac{1}{12\eta r}\frac{\partial}{\partial r}\left(rh^3\frac{\partial P}{\partial r}\right) \quad (4)$$

ΔP represents the pressure difference between the thin film at the protrusion and the thin film outside the immediate vicinity of the protrusion. We assume that the pressure difference is dominated by interfacial forces, $\Delta P \approx \Pi(h)$ where $\Pi(h)$ is the disjoining pressure due to interfacial forces. Away from the protrusion, we assume that $\Pi(h(B) = h + \Delta h)$ is negligible since the film thickness exceeds a few hundred nanometres (Figure 4e, Supplementary Figure 7).[23] The disjoining pressure due to interfacial forces can be approximated by:

$$\Pi(h) \approx \Pi_{\mathrm{vdW}} + \Pi_{\mathrm{EDL}} + \Pi_{\mathrm{st}} \quad (5)$$

The DLVO theory states that disjoining pressure $\Pi(h)$ is the sum of van der Waals (vdW) dispersion forces and electrical double layer (EDL) forces. In this case, Π_{EDL} is generally repulsive (negative) while Π_{vdW} can be either attractive or repulsive (depending on the interacting surfaces). Π_{st} is the contribution from the steric hindrance induced by free chains within the thin film. Due to the complex nature of beer, the presence of food proteins and further surface-active molecules (measured at 4.5 w/w percent) induces steric hindrance within the thin film during physical confinement. We know from DLS measurements (Supplementary Figure 10) that beer is composed of a polydisperse range of proteins and

particulates (at least 1 to 200 nm). Therefore, the steric contribution cannot be calculated. However, protein adsorption will induce film stabilization. Most likely, proteins are not permanently adsorbed at the beer-gas nor at the beer-protrusion interface, as the film eventually ruptures. Furthermore, irreversible adsorption could lead to the loss of performance after wetting. During drainage, proteins could move into the bulk liquid phase. Therefore, the superamphiphobic surface is still able to rupture bubbles while retaining functionality. In conclusion, the dynamic presence of steric hindrance contributions is significant and cannot be ignored.

To estimate how long film thinning would take without the presence of steric interactions we calculated film thinning using the Stokes-Reynold equation.

$$\frac{d}{dt}\frac{1}{h^2} = \frac{1}{3\eta r^2}(\Pi_{\mathrm{vdW}} + \Pi_{\mathrm{EDL}}) \quad (6)$$

We assume that the bubble-beer interface can be approximated to be flat close to the nano-protrusion $\left(\frac{dh}{dr} \approx 0\right)$, where h is the height of the film on the protrusion.[24] To estimate the van der Waals and electrostatic contribution to film thinning, we also estimate Π_{vdW} and Π_{EDL}.

5. Van der Waals Disjoining Pressure (Π_{vdW})

The disjoining pressure (Π_{vdW}) in the thin film, which dominates at low h, is represented by:

$$\Pi_{\mathrm{vdW}} = A_{\mathrm{H}}/6\pi h^3 \quad (7)$$

The Hamaker constant for the protrusion-induced film destabilization can be approximated as Fluoro(*i.e.* perfluorooctane)-Water-Air (FWA), $A_{\mathrm{H,FWA}} = +1.6 \times 10^{-20}$ J (attractive).[23] However, Hamaker interactions in liquid films of different ionic strengths may experience attractive-to-repulsive transitions.[25] In this case, a 34 mM solution of liquid at pH 4 (beer) interfacing a fluoro-functionalized hydrophobic surface should still exhibit overall attractive vdW interactions. These attractive interactions partially attribute to film thinning.

6. Electrical Double Layer Stabilization (Π_{EDL})

The stabilizing effect induced by the electrical double layer is approximated[26] by,

$$\Pi_{\mathrm{EDL}} = \Pi_0 exp(-\kappa h) \quad (8)$$

When modelled under a simple 1:1 electrolyte, $\Pi_0 = 64kT\rho_\infty tanh^2(e\Psi_0/4kT)$ is the "surface potential" and κ is the Debye-Hückel parameter where $\kappa = (2\rho_\infty e^2/\varepsilon\varepsilon_0 kT)^{1/2}$ whose inverse Debye length gives the exponential decay length of this disjoining pressure component Π_{EDL}. k is the Boltzmann constant, T is temperature, e is the electronic charge, Ψ_0 is the surface potential, ε is the dielectric constant of the electrolyte (approximated as water), ε_0 is the permittivity of free space, $\rho_\infty = 2000N_{\mathrm{A}}c$ represents the concentration expressed in ions.m^{-3}, with N_{A} being the Avogadro number and c the electrolyte concentration in mol.L^{-1}.[27] The electrolyte concentration (c) and surface potential (Ψ_0) of the dominantly aqueous beer is approximated at 34 mM[28] and -450 mV[29] respectively, giving a Debye length of 1.2 nm. Beer has a pH of 4. In this window of ion concentration and pH, liquid films have been shown to exhibit a repulsive electrostatic interaction.[25] However, in contrast to bulk foam (in air), bursting of the bubble's liquid film is more likely because the liquid film is continuously pressed against fluorinated protrusions (hydrophobic interactions). This potentially reduces the influence of electrostatic stabilization in the overall scheme of defoaming.

7. Film Thinning due to van der Waals (Π_{vdW}) and Electrostatics (Π_{EDL})

Supplementary Equation 6 can be infinitesimally defined (time-discretized) as:

$$h_{i+1} = \sqrt{\frac{3\eta r^2}{(\Pi_{\mathrm{vdW}} + \Pi_{\mathrm{EDL}})\,(\mathrm{d}t) + 3h_i^{-2}\eta r^2}} \quad (9)$$

where r is the size of the protrusion, dt as unit time, η as liquid viscosity, h_{i+1} and h_i are the time-step dependent thickness of the liquid film between the bubble and the particle. When solving Supplementary Equation 9, we estimate that it takes in the order of 3 ms to 300 ms

(range of r = 30 nm to r = 300 nm) for a beer film to sufficiently thin such that rupture occurs under the action of interfacial forces. We estimate that the film ruptures at a thickness of 150 ± 80 nm (at protrusions) based on the last frames captured in holographic microscopy (Supplementary Figure 11). We measured rupture times between 1 ms and 500 ms (Figure 4a-b). It is likely that the size of effective protrusions are $<$ 300 nm, but the drainage dynamics are further affected (slowed) by a spatial dependence on steric repulsion.

Supplementary References

1. Schellenberger, F. *et al.* Direct Observation of Drops on Slippery Lubricant-Infused Surfaces. *Soft Matter* **11**, 7617-7626 (2015).
2. Neugrodda, C., Gastl, M. & Becker, T. Comparison of Foam Analysis Methods and the Impact of Beer Components on Foam Stability. *J. Am. Soc. Brew. Chem.* **73**, 170-178 (2015).
3. Meyer, E. E., Rosenberg, K. J. & Israelachvili, J. Recent Progress in Understanding Hydrophobic Interactions. *PNAS* **103**, 15739 (2006).
4. Zhao, H., Law, K.-Y. & Sambhy, V. Fabrication, Surface Properties, and Origin of Superoleophobicity for a Model Textured Surface. *Langmuir* **27**, 5927-5935 (2011).
5. Aveyard, R., Cooper, P., Fletcher, P. D. I. & Rutherford, C. E. Foam Breakdown by Hydrophobic Particles and Nonpolar Oil. *Langmuir* **9**, 604-613 (1993).
6. Garrett, P. R. The Effect of Polytetrafluoroethylene Particles on the Foamability of Aqueous Surfactant Solutions. *J. Colloid Interface Sci.* **69**, 107-121 (1979).
7. Wong, W. S. Y. *et al.* Omnidirectional Self-Assembly of Transparent Superoleophobic Nanotextures. *ACS Nano* **11**, 587–596 (2016).
8. Smith, J. D. *et al.* Droplet Mobility on Lubricant-Impregnated Surfaces. *Soft Matter* **9**, 1772-1780 (2013).
9. Teisala, H. *et al.* Wetting over Pre-Existing Liquid Films. *Phys. Rev. Fluids* **3**, 084002 (2018).
10. Sun, J. & Weisensee, P. B. Microdroplet Self-Propulsion During Dropwise Condensation on Lubricant-Infused Surfaces. *Soft Matter* **15**, 4808-4817 (2019).
11. Pugh, R. J. Foaming, Foam Films, Antifoaming and Defoaming. *Adv. Colloid Interface Sci.* **64**, 67-142 (1996).
12. Robinson, J. V. & Woods, W. W. A Method of Selecting Foam Inhibitors. *J. Soc. Chem. Ind.* **67**, 361-365 (1948).
13. Scheludko, A. Über Das Ausfließen Der Lösung Aus Schaumfilmen. *Kolloid-Zeitschrift* **155**, 39-44 (1957).
14. Liu, B. *et al.* Coalescence of Bubbles with Mobile Interfaces in Water. *Phys. Rev. Lett.* **122**, 194501 (2019).
15. Manica, R., Klaseboer, E. & Chan, D. Y. C. The Hydrodynamics of Bubble Rise and Impact with Solid Surfaces. *Adv. Colloid Interface Sci.* **235**, 214-232 (2016).
16. Irajizad, P., Nazifi, S. & Ghasemi, H. Icephobic Surfaces: Definition and Figures of Merit. *Adv. Colloid Interface Sci.* **269**, 203-218 (2019).
17. Reynolds, O. On the Theory of Lubrication and Its Application to Mr. Beauchamp Tower's Experiments, Including an Experimental Determination of the Viscosity of Olive Oil. *Philos. Trans. Royal Soc.* **177**, 157-234 (1886).

18 Vrij, A. & Overbeek, J. T. H. G. Rupture of Thin Liquid Films Due to Spontaneous Fluctuations in Thickness. *J. Am. Chem. Soc.* **90**, 3074-3078 (1968).
19 Chan, D. Y. C., Klaseboer, E. & Manica, R. Film Drainage and Coalescence between Deformable Drops and Bubbles. *Soft Matter* **7**, 2235-2264 (2011).
20 Batchelor, G. K. *An Introduction to Fluid Dynamics*. (Cambridge University Press, 2000).
21 Leal, L. G. *Advanced Transport Phenomena: Fluid Mechanics and Convective Transport Processes*. (Cambridge University Press, 2007).
22 Cascão Pereira, L. G., Johansson, C., Radke, C. J. & Blanch, H. W. Surface Forces and Drainage Kinetics of Protein-Stabilized Aqueous Films. *Langmuir* **19**, 7503-7513 (2003).
23 Butt, H. J. & Kappl, M. *Surface and Interfacial Forces*. (Wiley, 2018).
24 Baumli, P. *et al.* Flow-Induced Long-Term Stable Slippery Surfaces. *Adv. Sci.* **6**, 1900019 (2019).
25 Tabor, R. F. *et al.* Homo- and Hetero-Interactions between Air Bubbles and Oil Droplets Measured by Atomic Force Microscopy. *Soft Matter* **7**, 8977-8983 (2011).
26 Israelachvili, J. N. *Intermolecular and Surface Forces: With Applications to Colloidal and Biological Systems*. (Academic Press, 1985).
27 Yaminsky, V. V., Ohnishi, S., Vogler, E. A. & Horn, R. G. Stability of Aqueous Films between Bubbles. Part 1. The Effect of Speed on Bubble Coalescence in Purified Water and Simple Electrolyte Solutions. *Langmuir* **26**, 8061-8074 (2010).
28 Ben, D. *et al.* Manipulations to the Alcohol and Sodium Content of Beer for Postexercise Rehydration. *Int. J. Sport. Nutr. Exe.* **25**, 262-270 (2015).
29 Titze, J., Christian, M., Jacob, F., Parlar, H. & Ilberg, V. The Possibilities of Particle Analysis Demonstrated by the Measurement of the Colloidal Stability of Filtered Beer. *J. Inst. Brew.* **116**, 405-412 (2010).
