# Supplementary material for: Super liquid repellent surfaces for anti-foaming and froth management
Source: Nat Commun. 2021 Sep 9;12:5358. doi: 10.1038/s41467-021-25556-w (PMC8429590; doi:10.1038/s41467-021-25556-w)
Supplement: Supplementary file 2 — Description of Additional Supplementary Files [file 41467_2021_25556_MOESM2_ESM.pdf]

Description of Additional Supplementary Files

File name: Supplementary Movie 1
Description: Superamphiphobic glass (SA) vs. Control glass pouring beer. Real-time 1X speed.

File name: Supplementary Movie 2
Description: Control glass vs. Liquid-like PDMS (LLPDMS) vs. Superamphiphobic Surface (SA) at 1 bar dispensing pressure, fast-forwarded 10X speed.

File name: Supplementary Movie 3
Description: SLIPS glasses: Hexadecane (HD) vs. Silicone oil, 5 cSt (SO-5cSt) vs. Silicone oil, 500 cSt (SO-500cSt) at 1 bar dispensing pressure, fast-forwarded 10X speed.

File name: Supplementary Movie 4
Description: Control glass vs. Silicone oil, 5 cSt (SO-5cSt) vs. Superamphiphobic Surface (SA) at 1 bar dispensing pressure, fast-forwarded 10X speed.

File name: Supplementary Movie 5
Description: Tracking algorithm for beer and foam dimension. 30s per frame. Beer and foam column: 5.5 cm x 12 cm.

File name: Supplementary Movie 6
Description: Macro-imaging of bubbles-on-interfaces. Control vs. SA vs. SLIPS-SO vs. SLIPS-HD. Real-time 1X speed. Area of 2 mm x 2 mm at 2.5 cm below the maximum foam line.

File name: Supplementary Movie 7
Description: Control vs. Superamphiphobic Defoamer (SABrush) at 1 bar dispensing pressure, fast-forwarded 10X speed.

File name: Supplementary Movie 8
Description: Anti-foaming behavior vs. dynamically growing C12E5 soap foams. Real-time 1X speed.

File name: Supplementary Movie 9
Description: Anti-foaming behavior vs. dynamically growing commercial soap foams. Real-time 1X speed.

File name: Supplementary Movie 10
Description: Tracking algorithm for bubbles-on-interfaces. Real-time 1X speed. Area of 2 mm x 2 mm at 2.5 cm below the maximum foam line.
