# Supplementary figures and images for: Super liquid repellent surfaces for anti-foaming and froth management
Source: Nat Commun. 2021 Sep 9;12:5358. doi: 10.1038/s41467-021-25556-w (PMC8429590; doi:10.1038/s41467-021-25556-w)

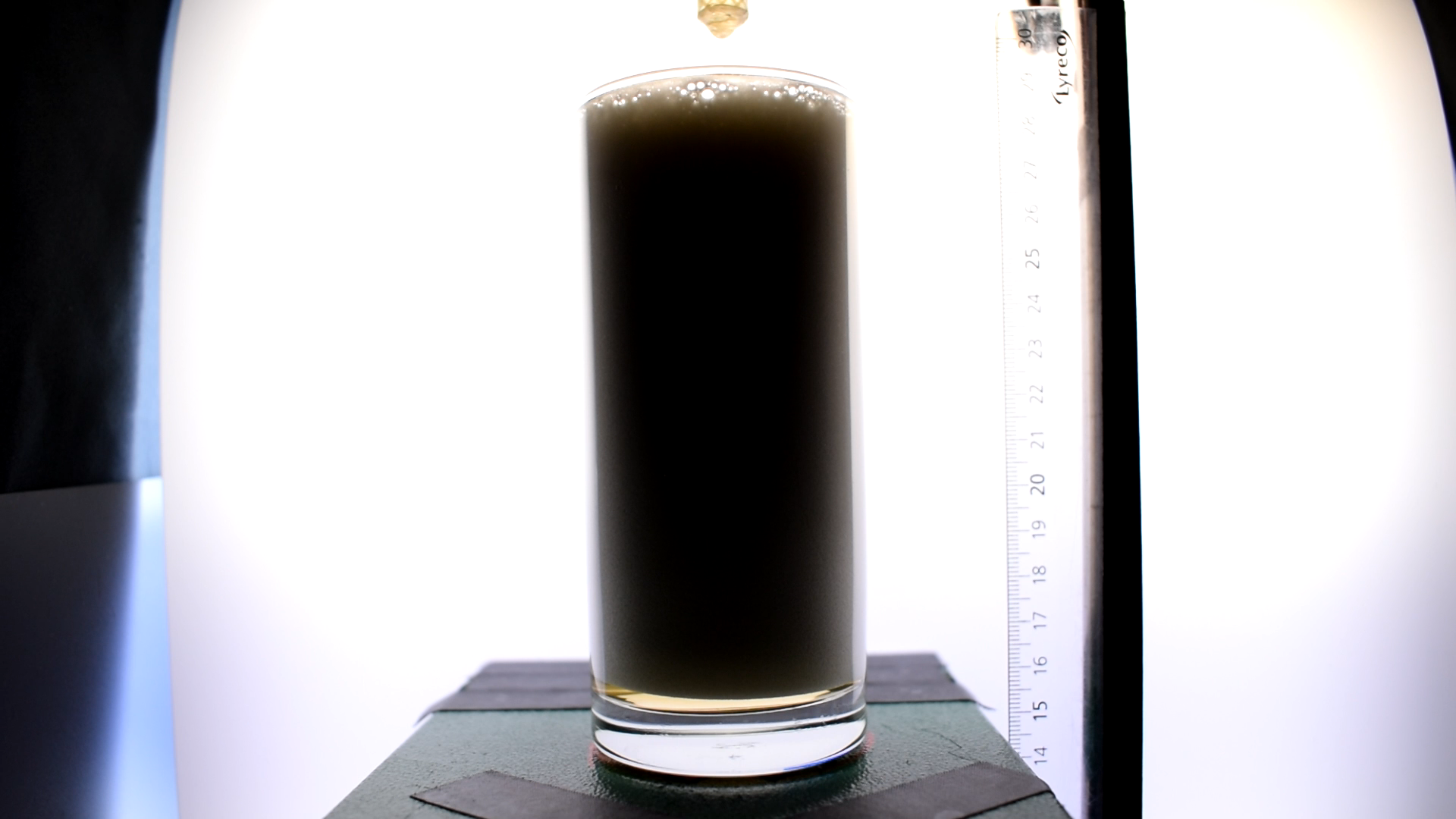

Supplement: Supplementary file 13 — Source Data [file 41467_2021_25556_MOESM13_ESM.zip › Supplementary Software/Controls/Control-01-Sample/01.bmp]

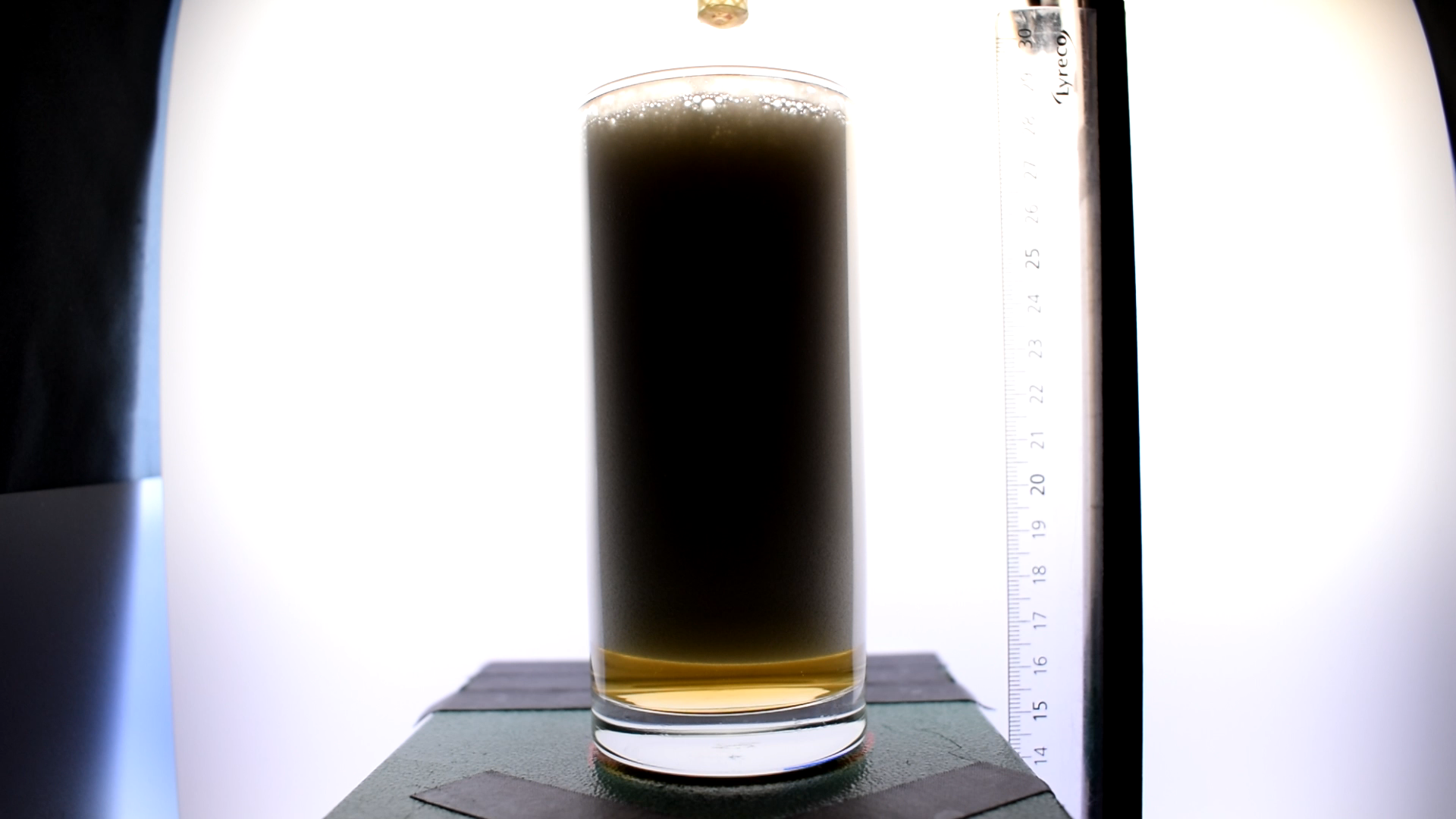

Supplement: Supplementary file 13 — Source Data [file 41467_2021_25556_MOESM13_ESM.zip › Supplementary Software/Controls/Control-01-Sample/02.bmp]

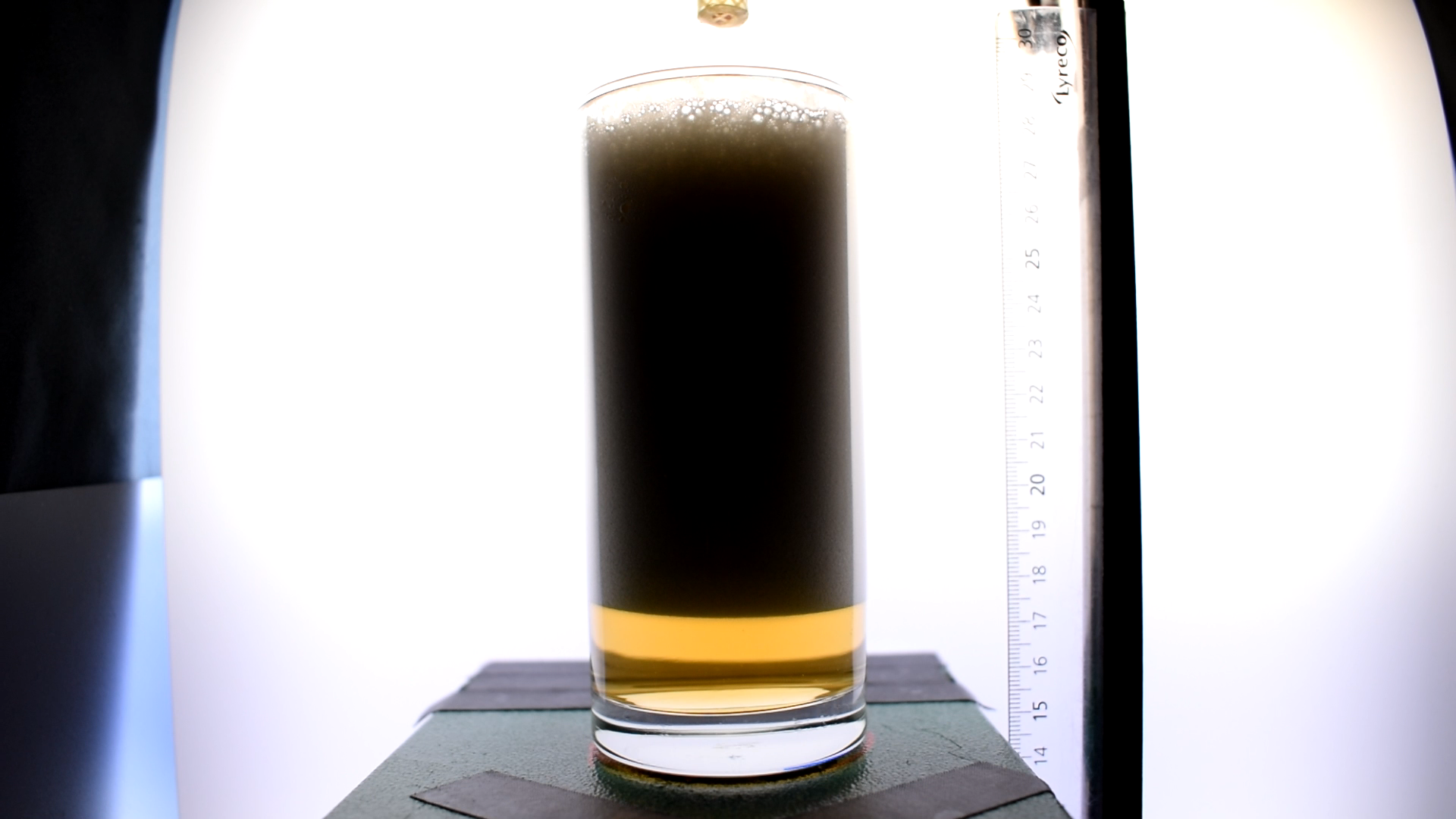

Supplement: Supplementary file 13 — Source Data [file 41467_2021_25556_MOESM13_ESM.zip › Supplementary Software/Controls/Control-01-Sample/03.bmp]

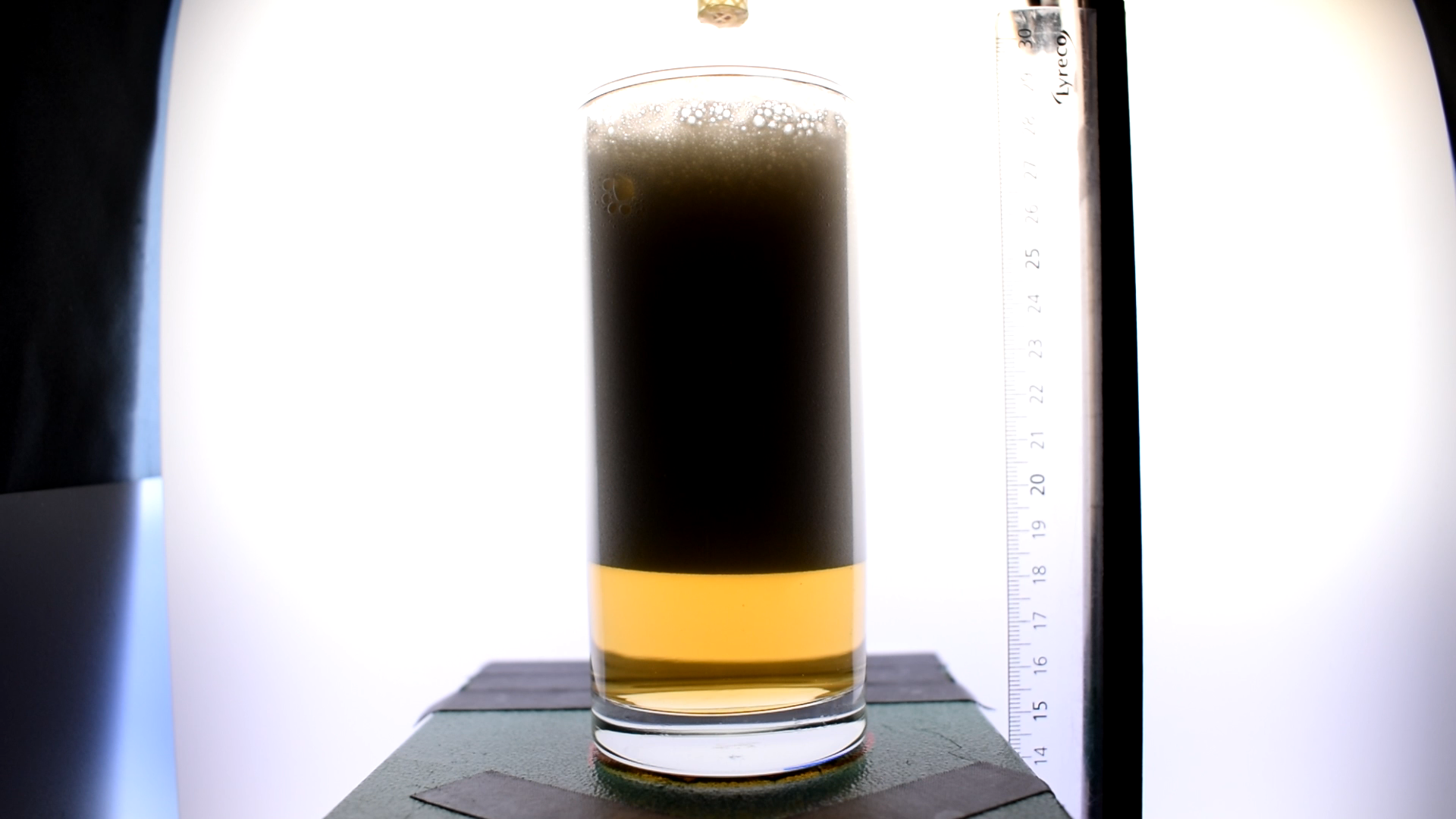

Supplement: Supplementary file 13 — Source Data [file 41467_2021_25556_MOESM13_ESM.zip › Supplementary Software/Controls/Control-01-Sample/04.bmp]

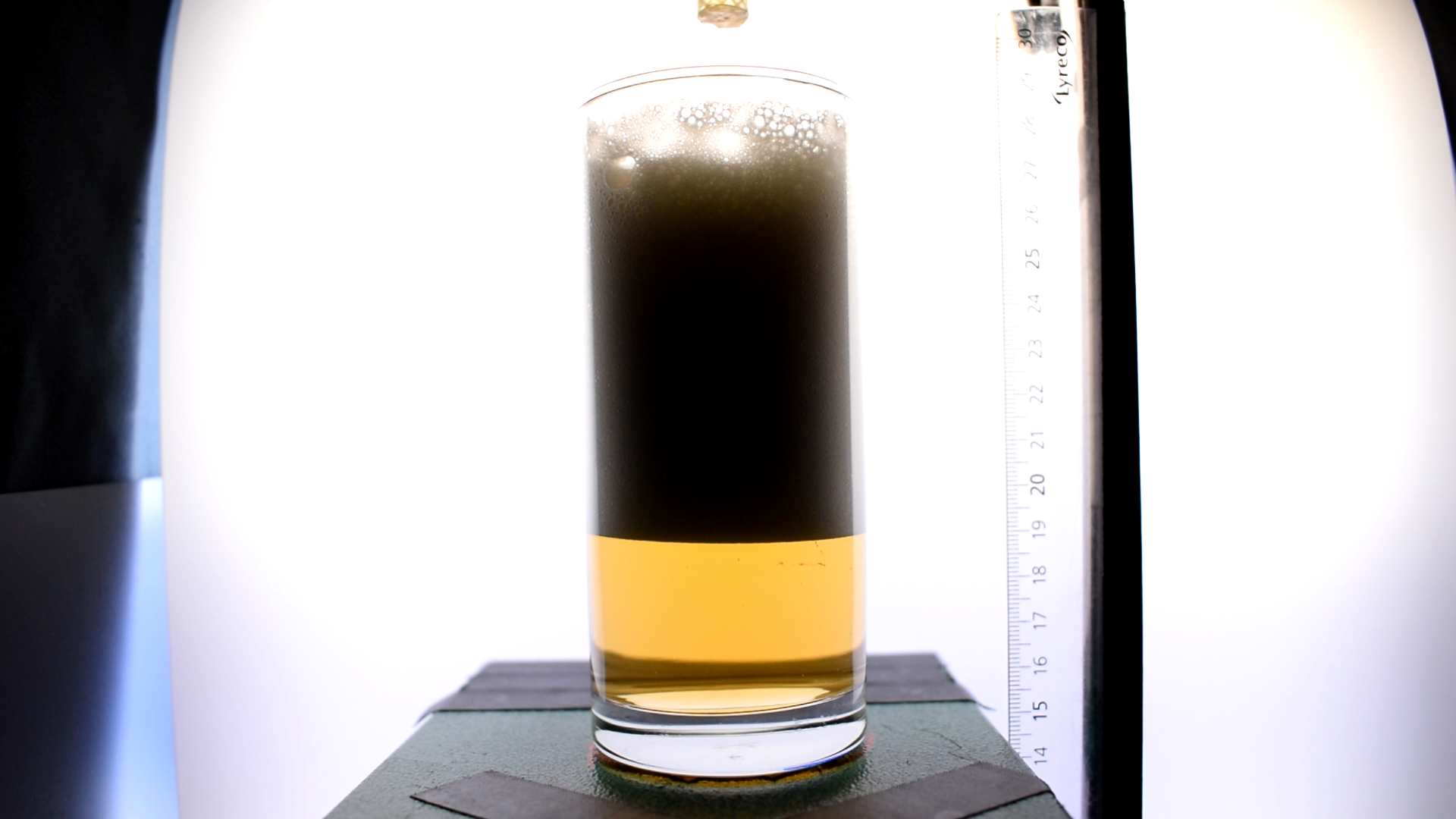

Supplement: Supplementary file 13 — Source Data [file 41467_2021_25556_MOESM13_ESM.zip › Supplementary Software/Controls/Control-01-Sample/05.bmp]

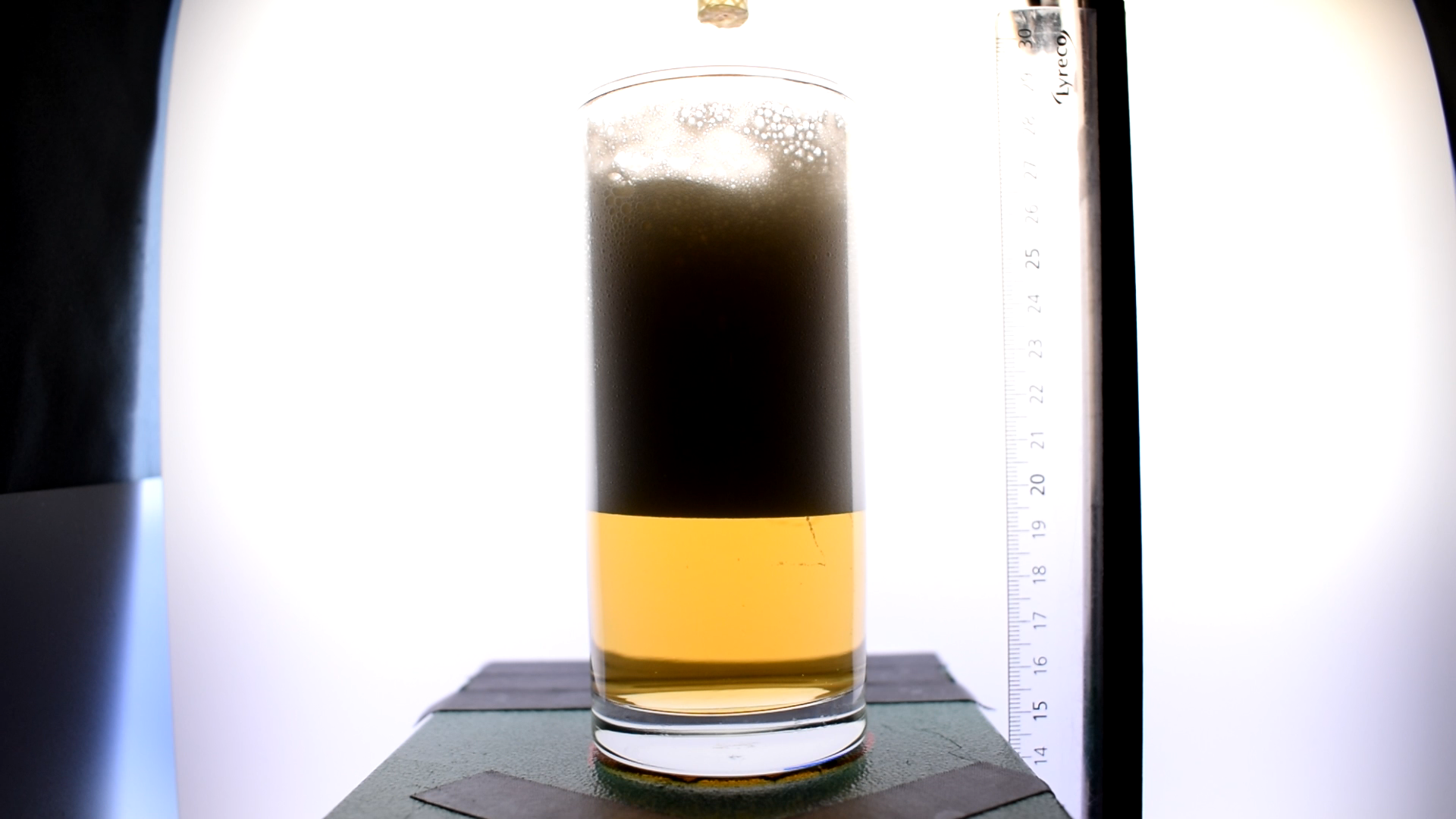

Supplement: Supplementary file 13 — Source Data [file 41467_2021_25556_MOESM13_ESM.zip › Supplementary Software/Controls/Control-01-Sample/06.bmp]

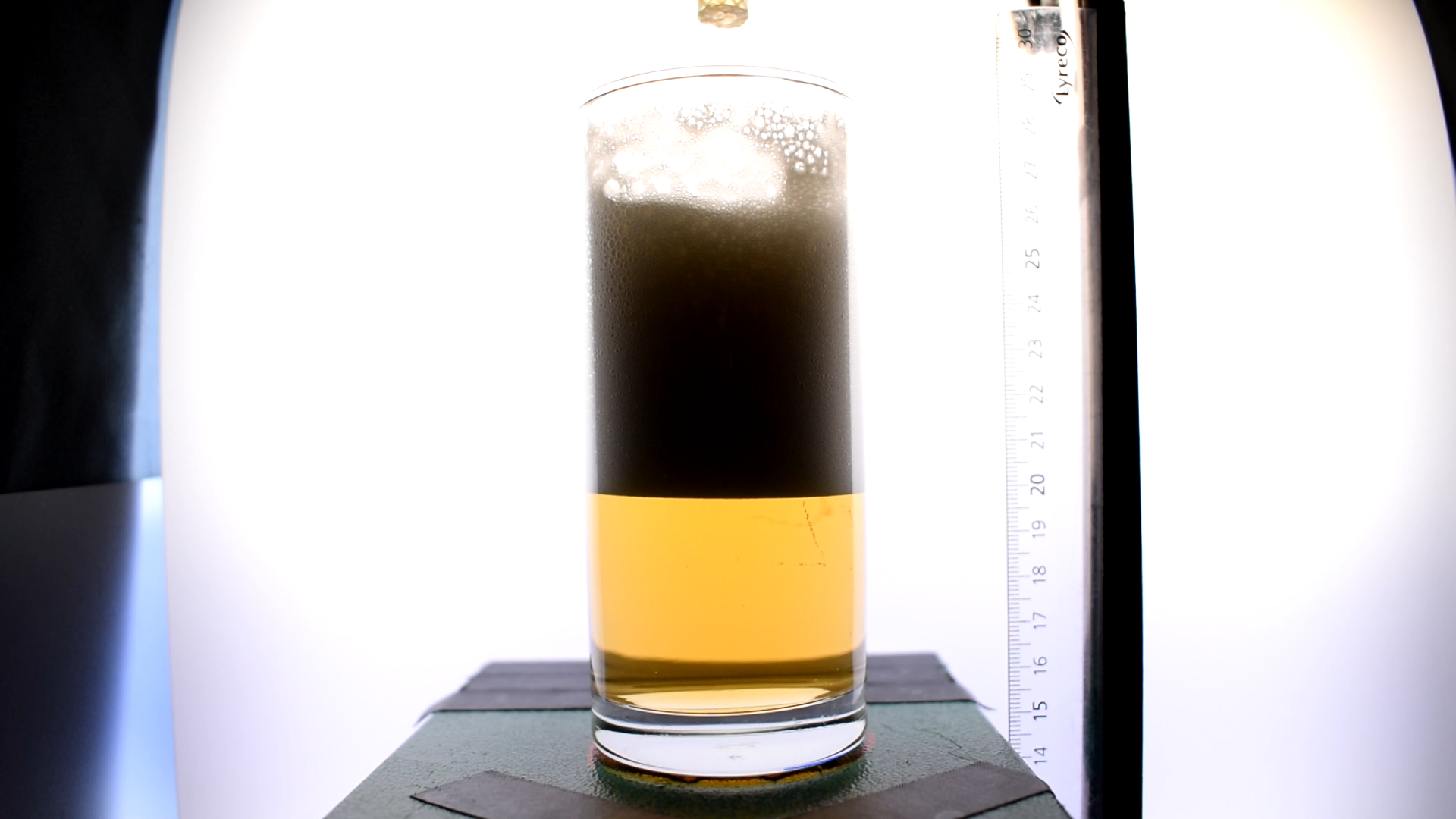

Supplement: Supplementary file 13 — Source Data [file 41467_2021_25556_MOESM13_ESM.zip › Supplementary Software/Controls/Control-01-Sample/07.bmp]

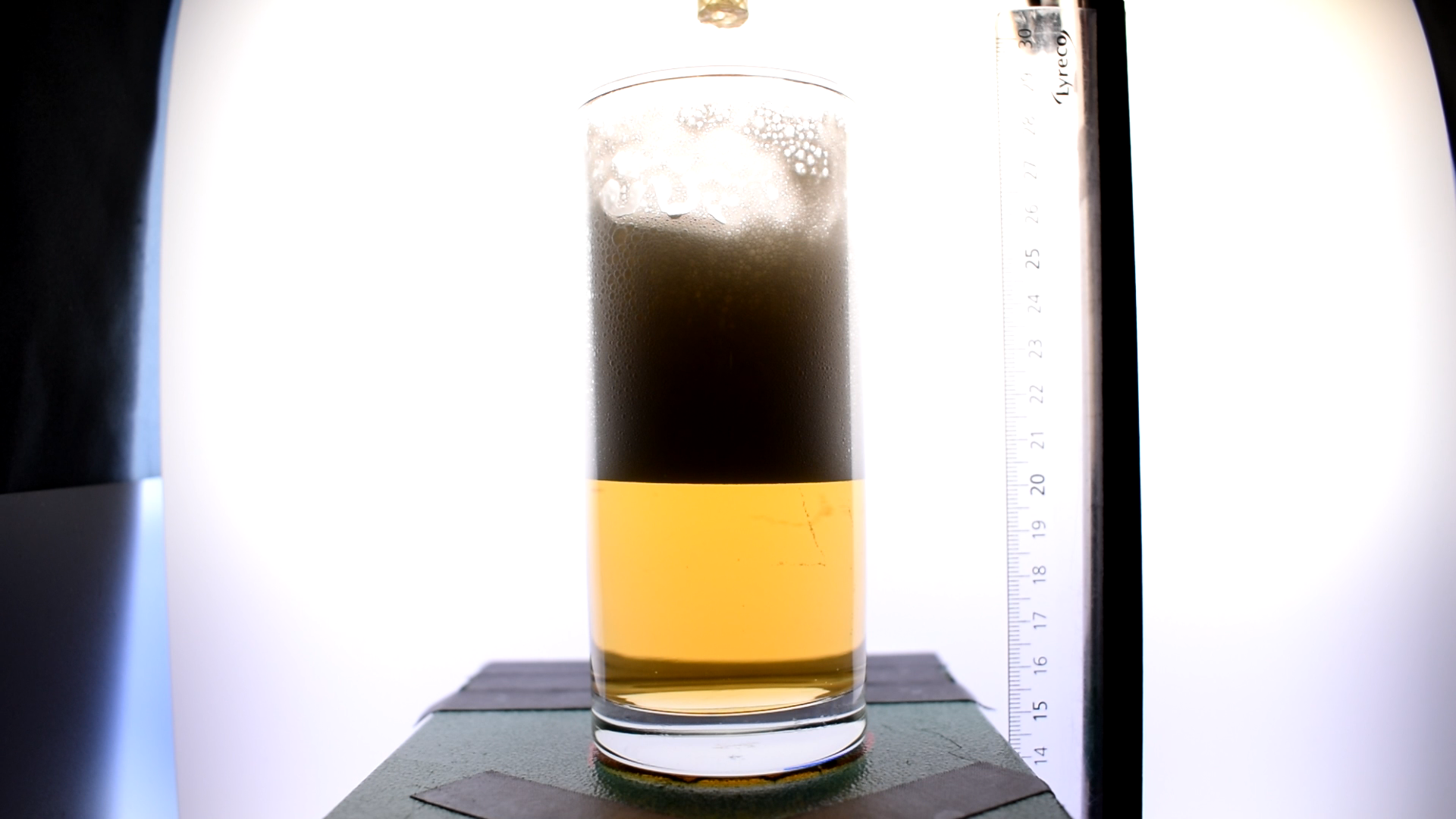

Supplement: Supplementary file 13 — Source Data [file 41467_2021_25556_MOESM13_ESM.zip › Supplementary Software/Controls/Control-01-Sample/08.bmp]

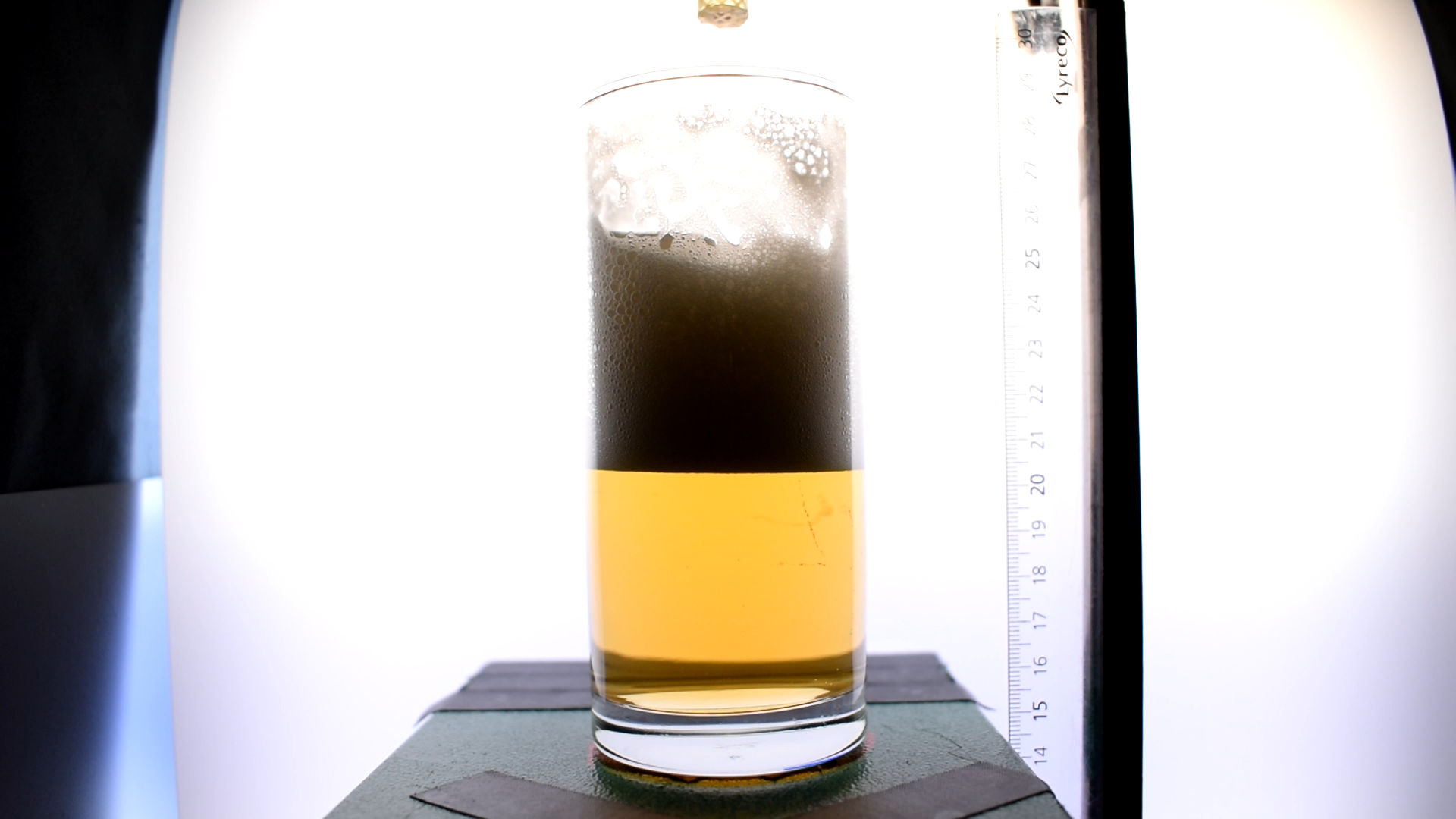

Supplement: Supplementary file 13 — Source Data [file 41467_2021_25556_MOESM13_ESM.zip › Supplementary Software/Controls/Control-01-Sample/09.bmp]

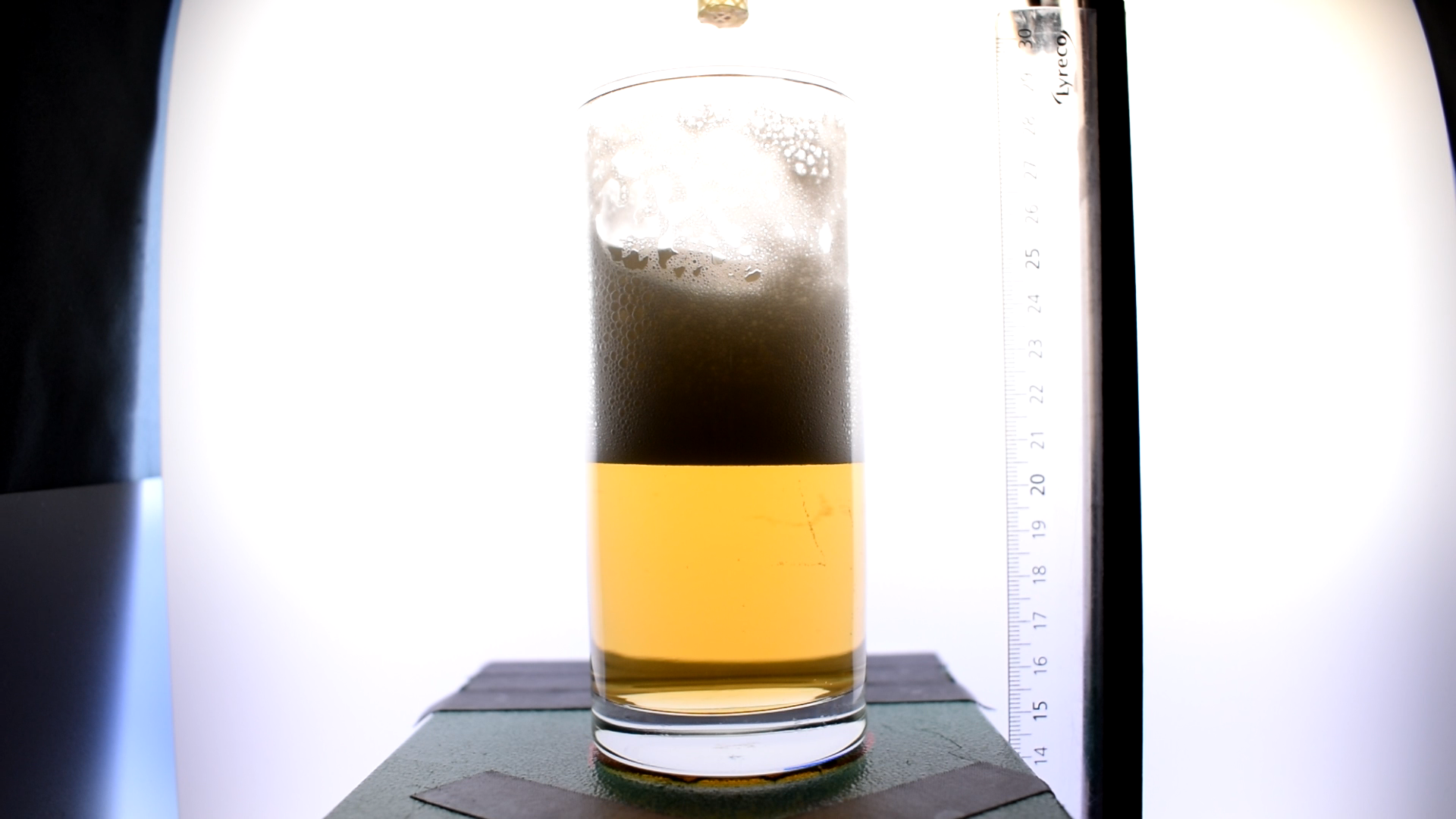

Supplement: Supplementary file 13 — Source Data [file 41467_2021_25556_MOESM13_ESM.zip › Supplementary Software/Controls/Control-01-Sample/10.bmp]

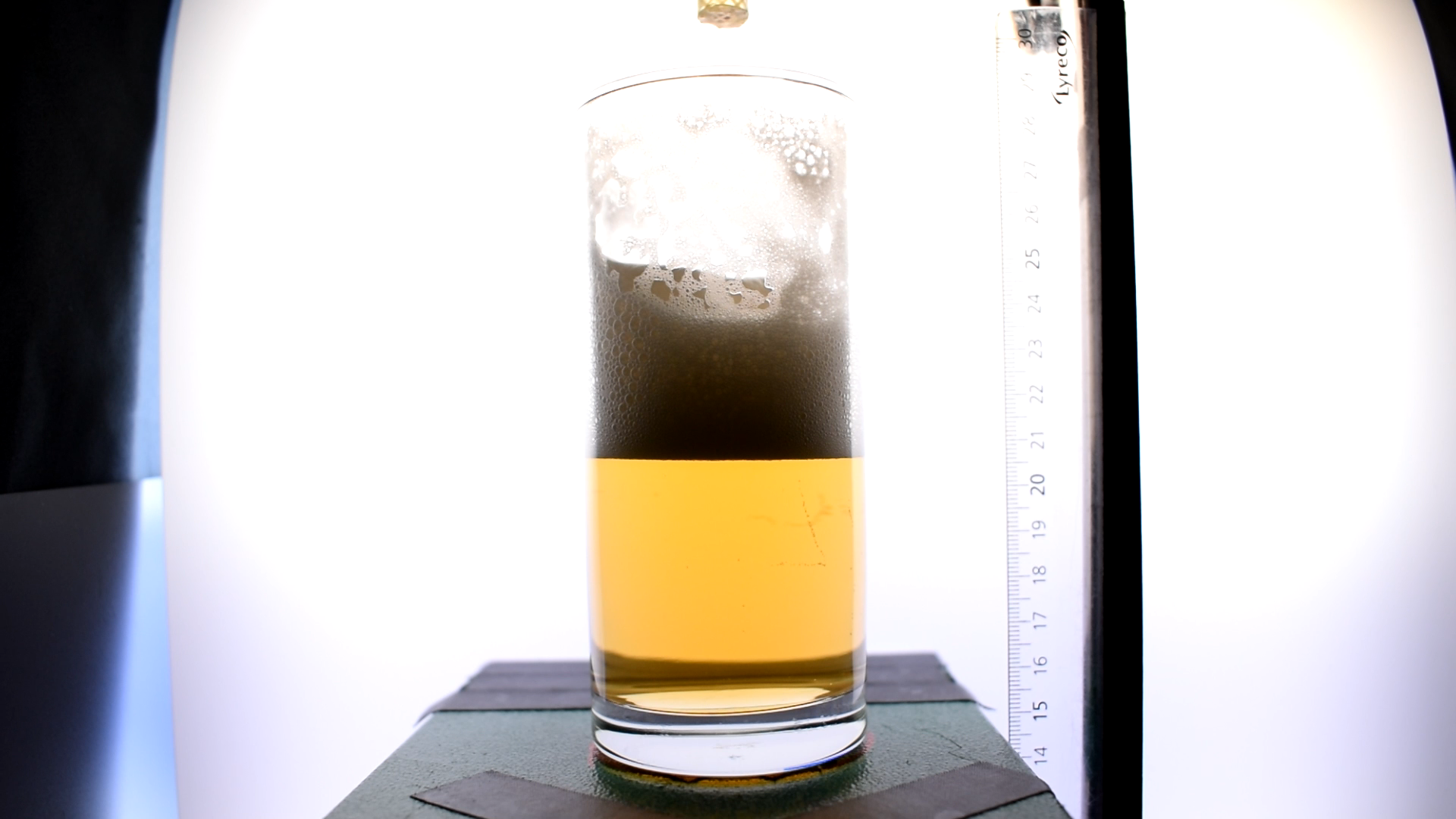

Supplement: Supplementary file 13 — Source Data [file 41467_2021_25556_MOESM13_ESM.zip › Supplementary Software/Controls/Control-01-Sample/11.bmp]

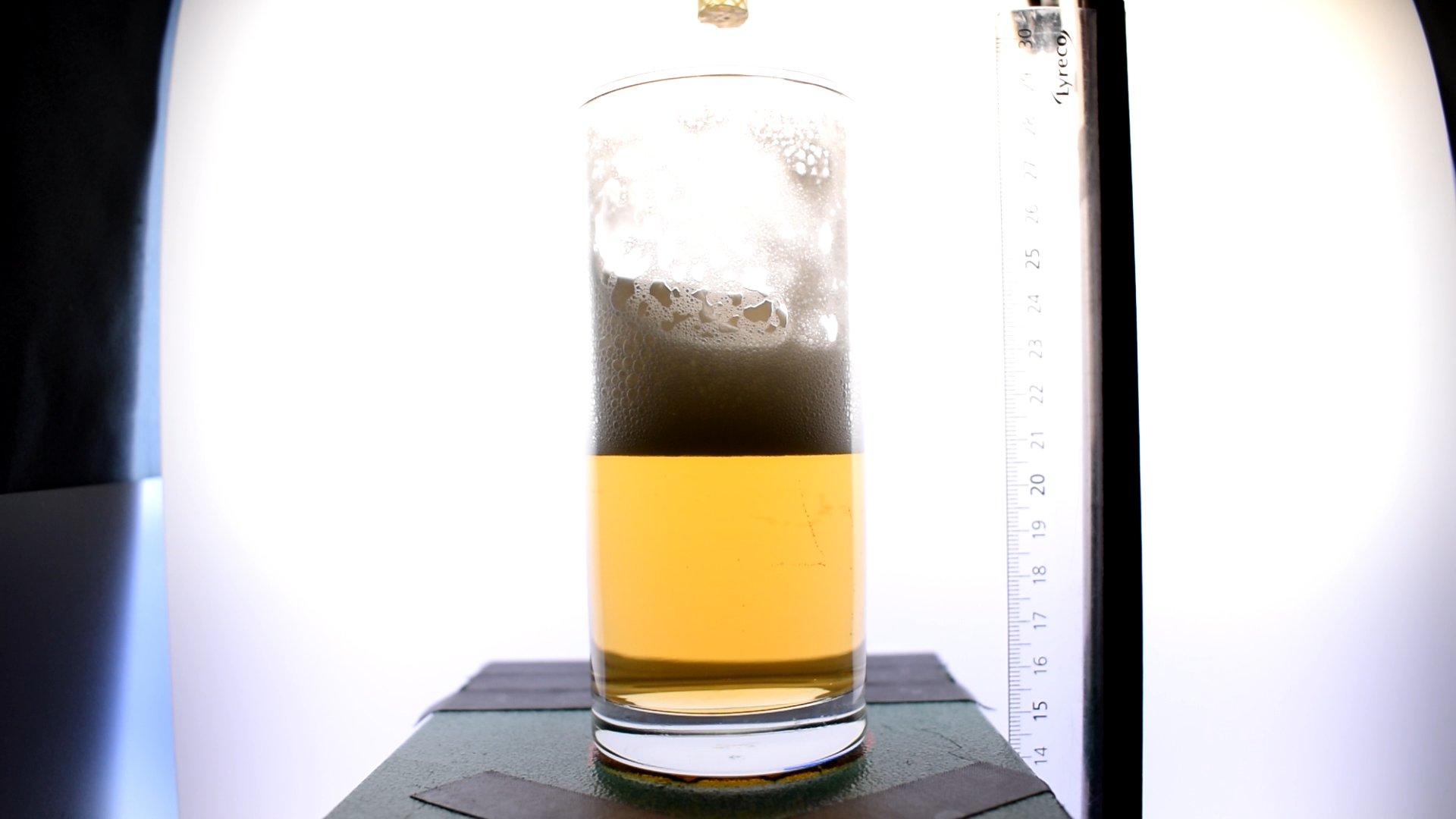

Supplement: Supplementary file 13 — Source Data [file 41467_2021_25556_MOESM13_ESM.zip › Supplementary Software/Controls/Control-01-Sample/12.bmp]

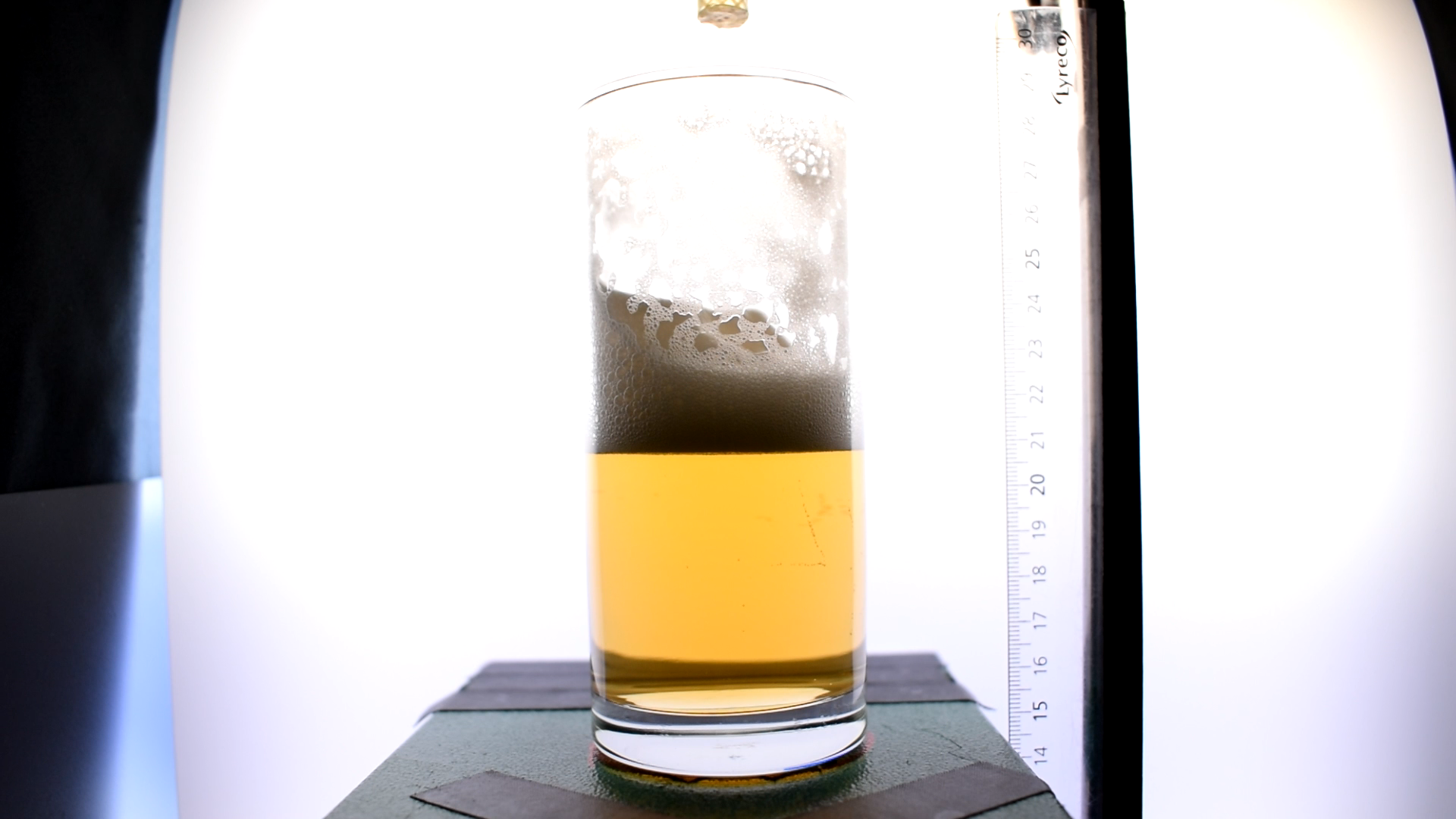

Supplement: Supplementary file 13 — Source Data [file 41467_2021_25556_MOESM13_ESM.zip › Supplementary Software/Controls/Control-01-Sample/13.bmp]

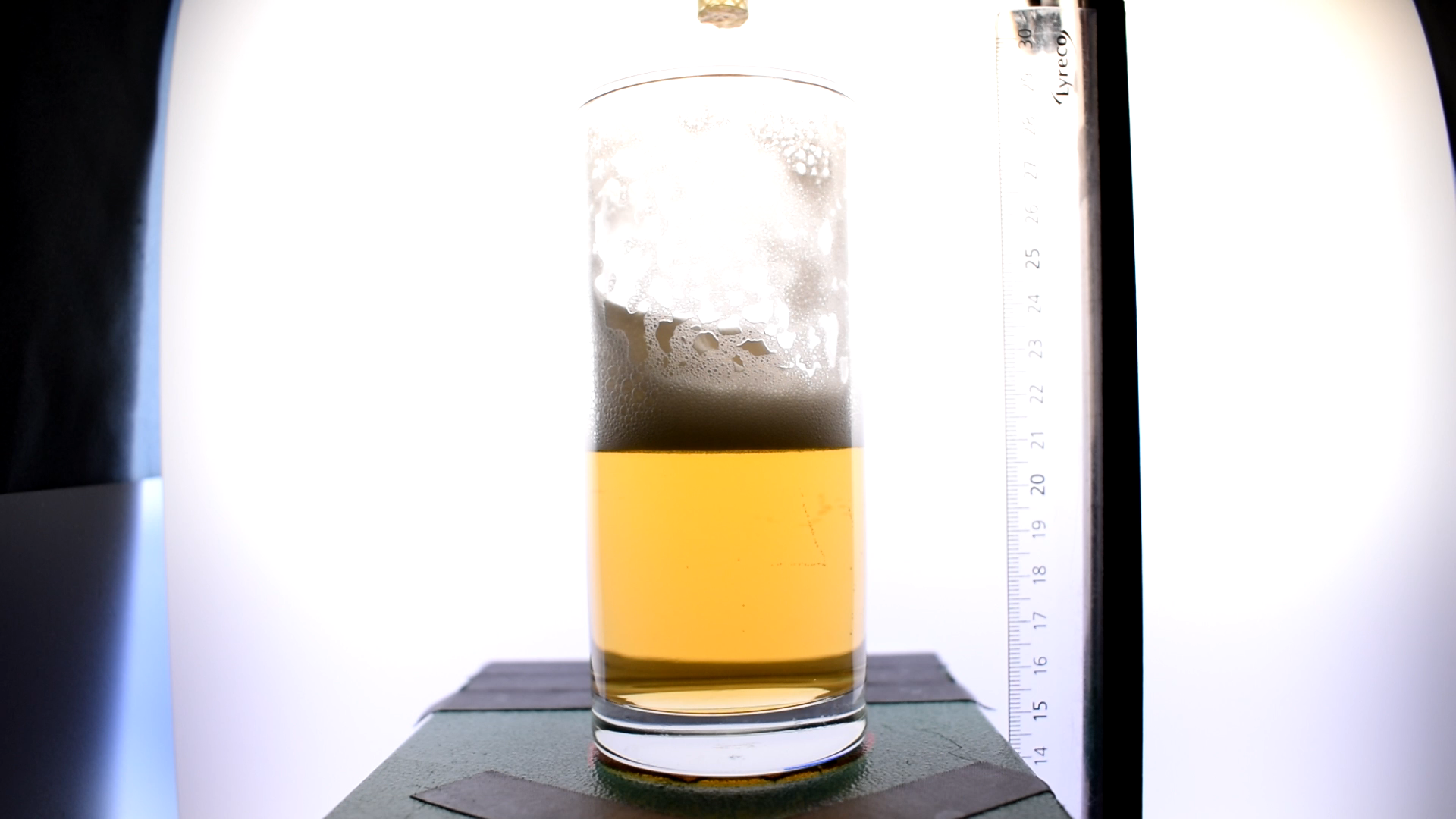

Supplement: Supplementary file 13 — Source Data [file 41467_2021_25556_MOESM13_ESM.zip › Supplementary Software/Controls/Control-01-Sample/14.bmp]

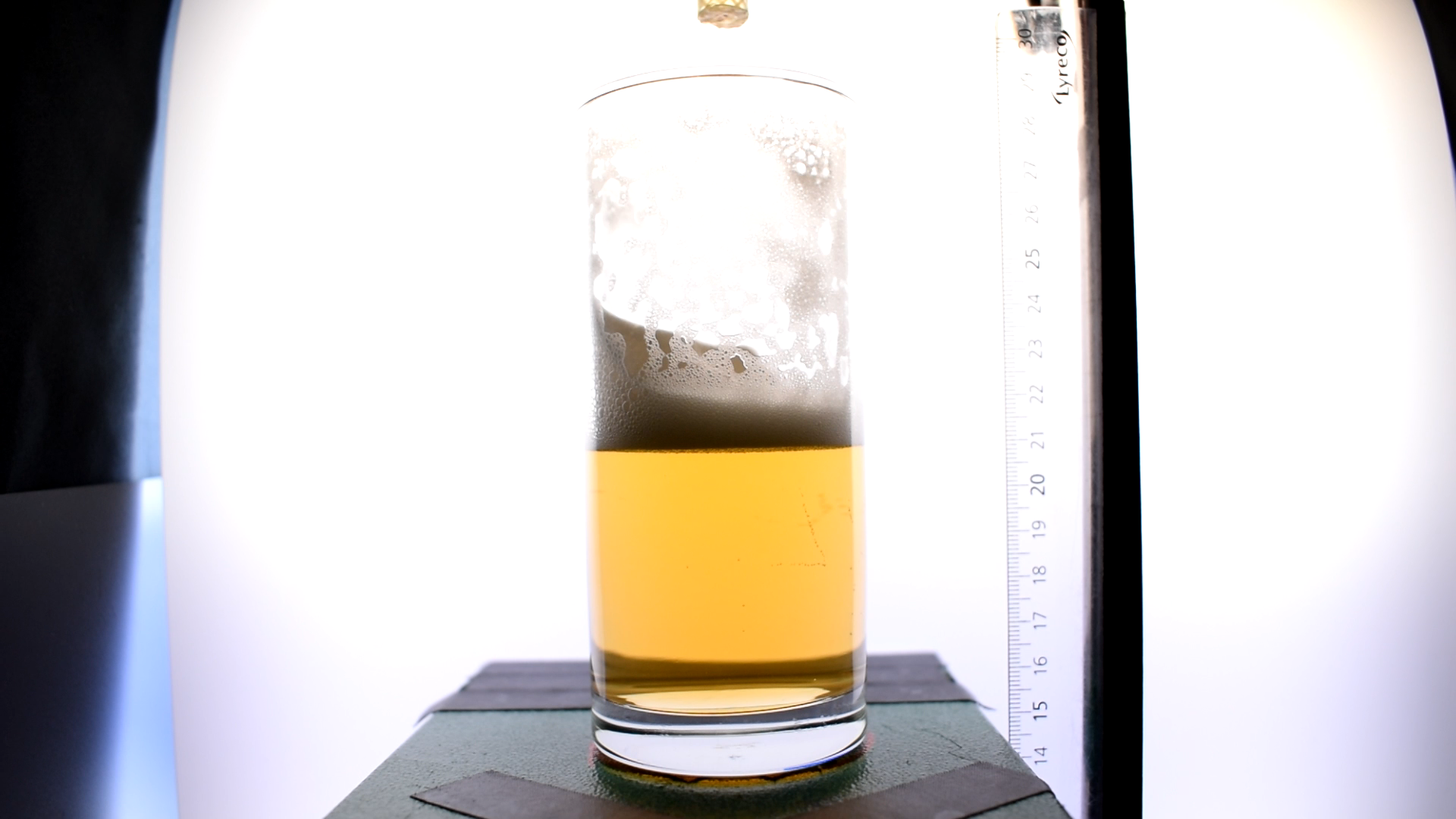

Supplement: Supplementary file 13 — Source Data [file 41467_2021_25556_MOESM13_ESM.zip › Supplementary Software/Controls/Control-01-Sample/15.bmp]

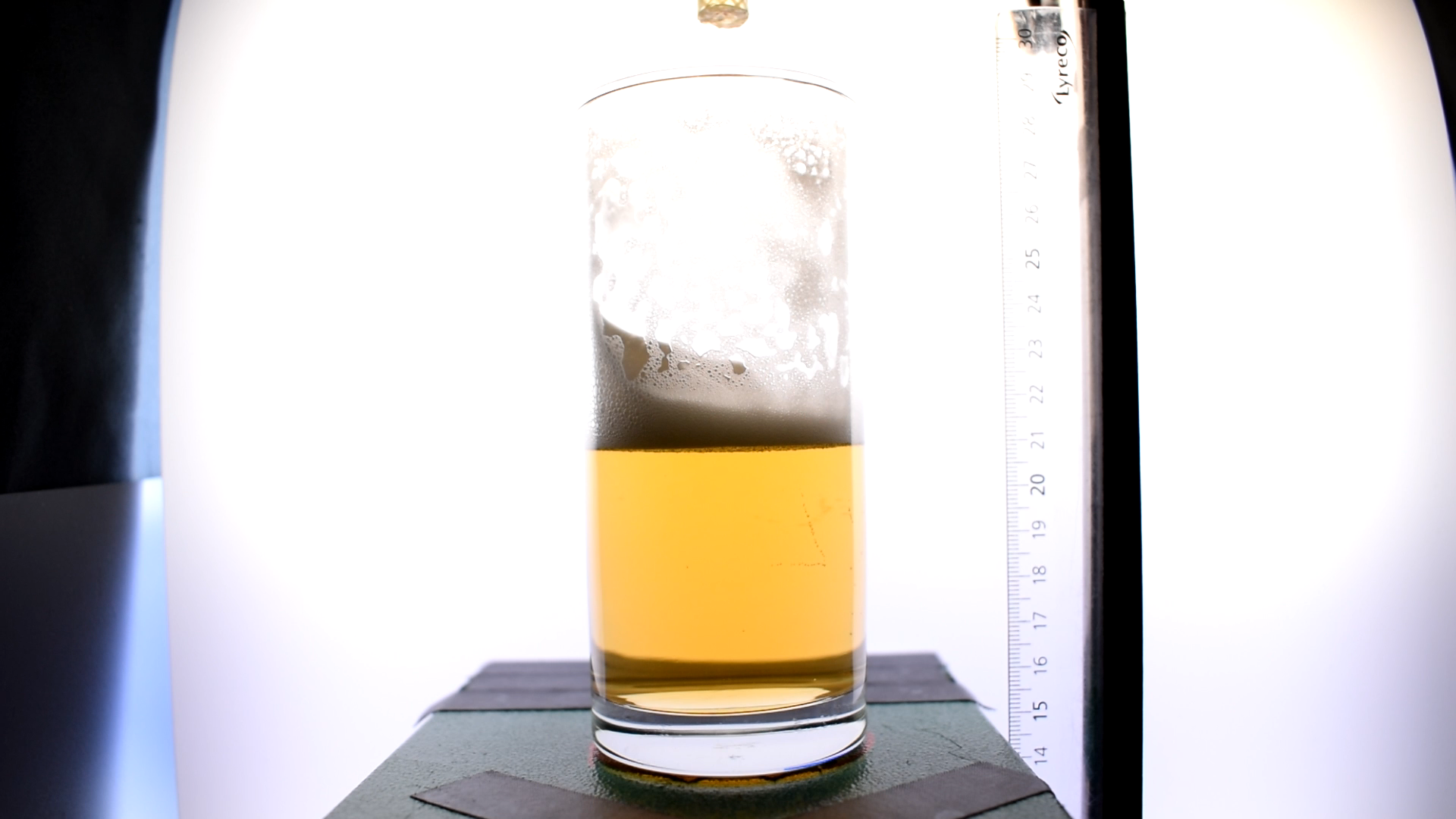

Supplement: Supplementary file 13 — Source Data [file 41467_2021_25556_MOESM13_ESM.zip › Supplementary Software/Controls/Control-01-Sample/16.bmp]

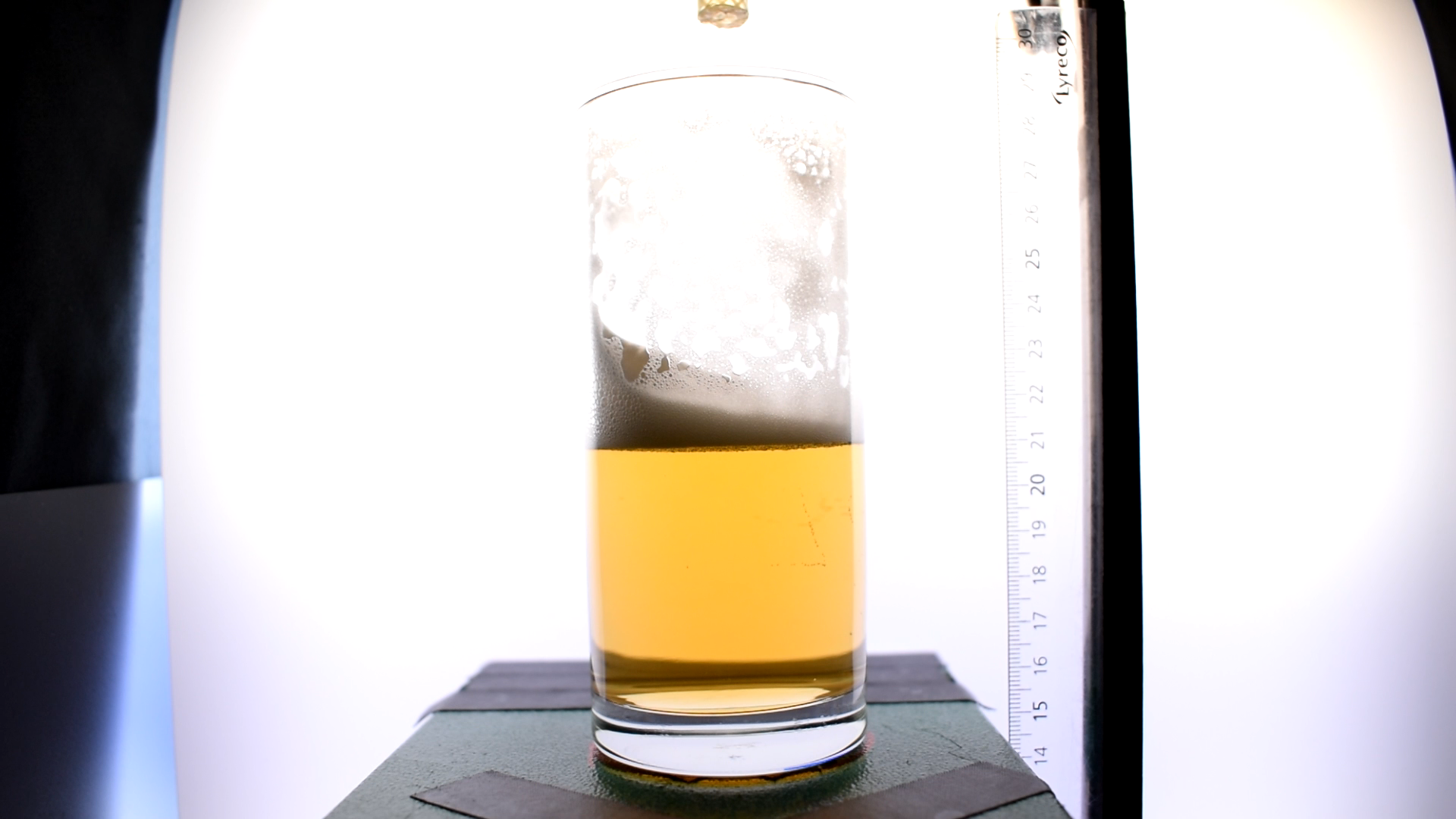

Supplement: Supplementary file 13 — Source Data [file 41467_2021_25556_MOESM13_ESM.zip › Supplementary Software/Controls/Control-01-Sample/17.bmp]

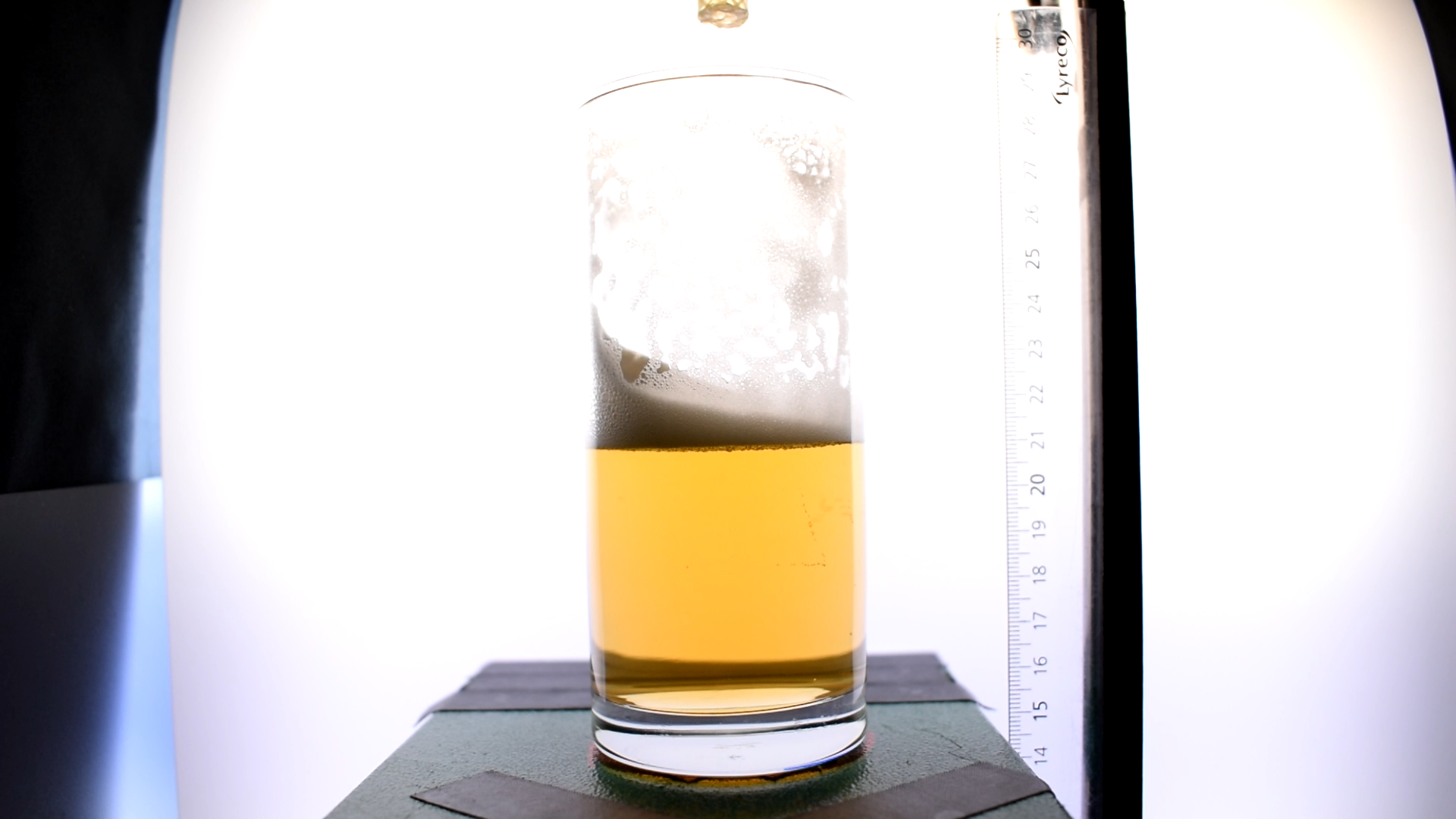

Supplement: Supplementary file 13 — Source Data [file 41467_2021_25556_MOESM13_ESM.zip › Supplementary Software/Controls/Control-01-Sample/18.bmp]

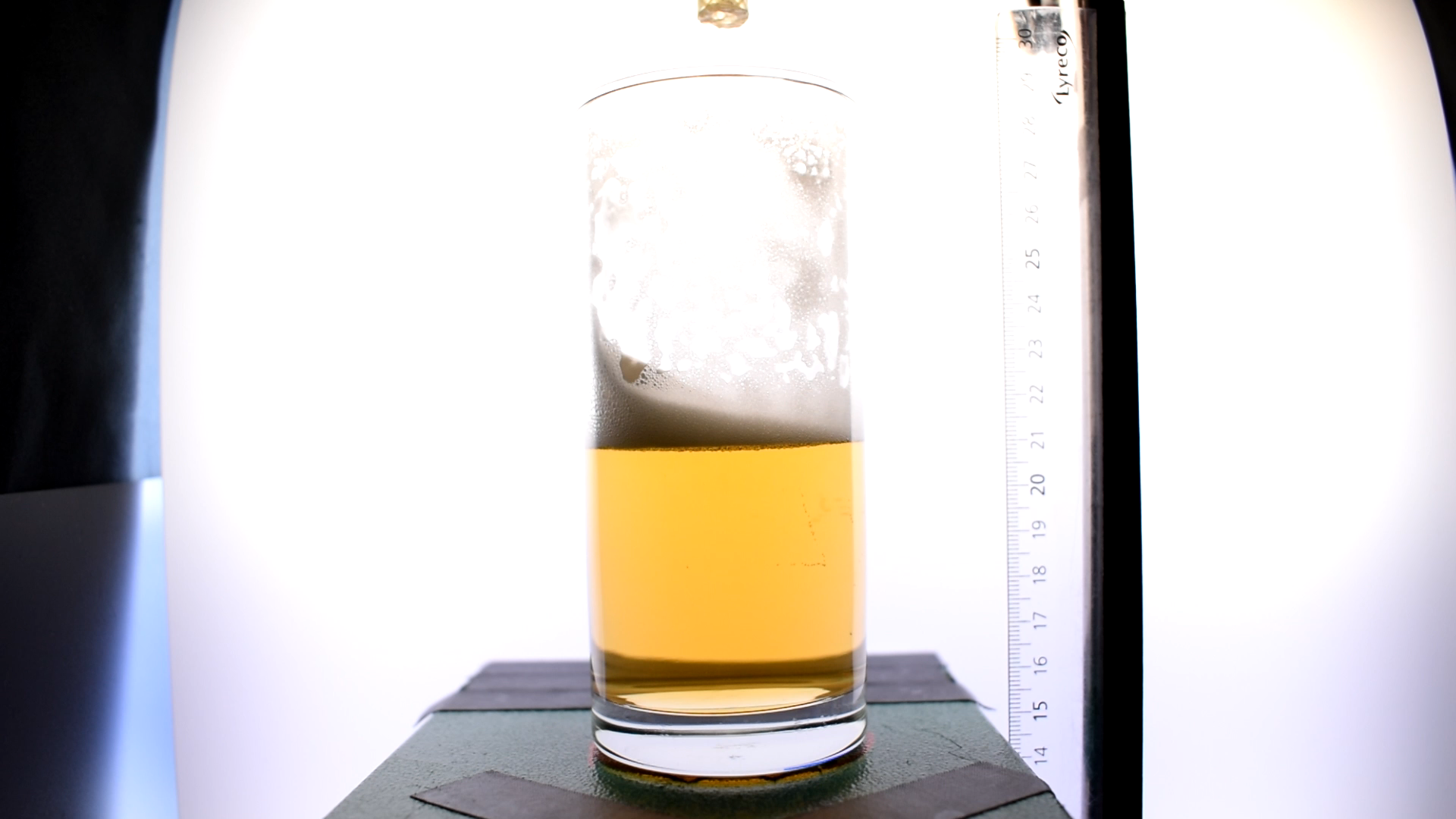

Supplement: Supplementary file 13 — Source Data [file 41467_2021_25556_MOESM13_ESM.zip › Supplementary Software/Controls/Control-01-Sample/19.bmp]

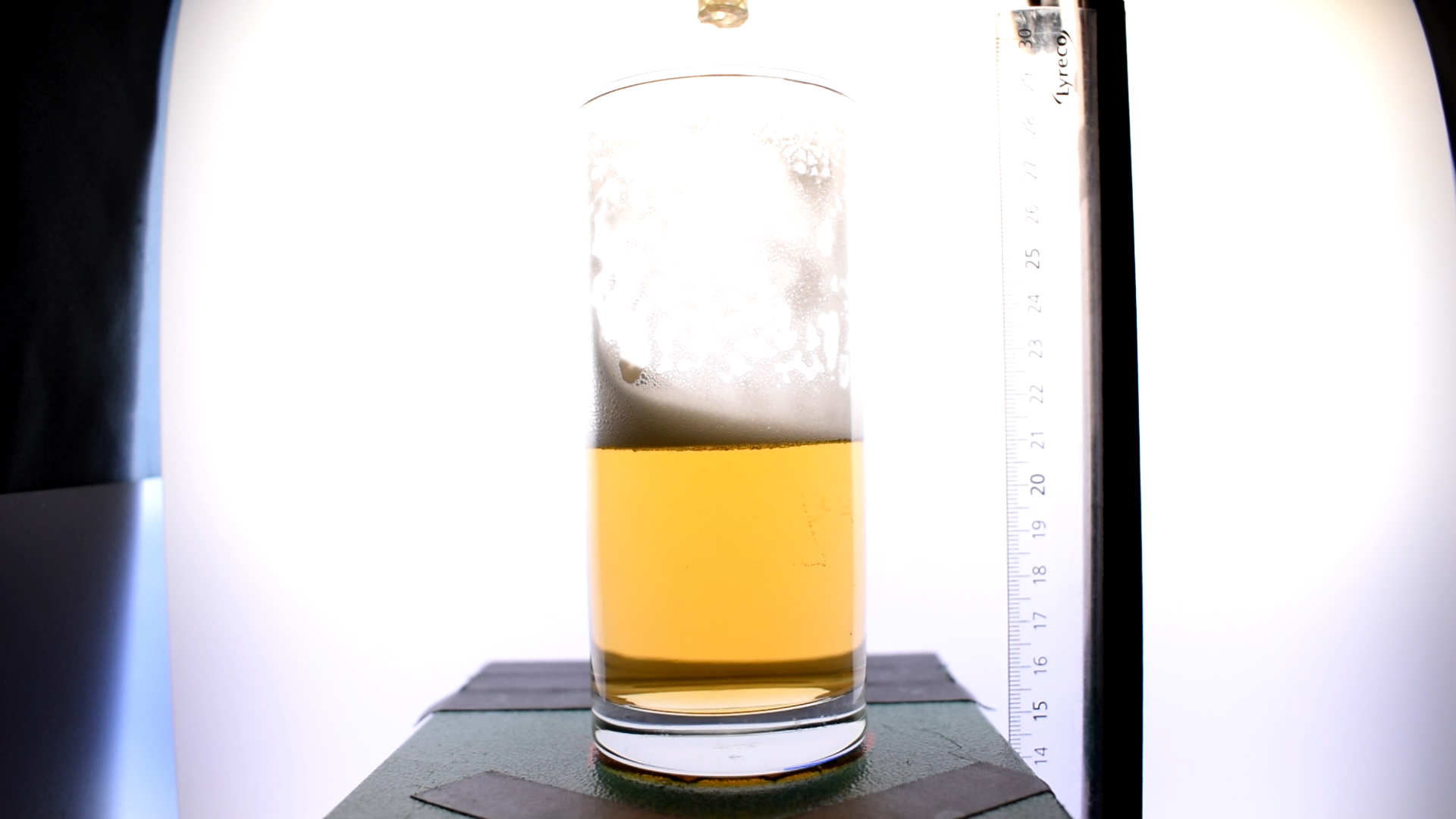

Supplement: Supplementary file 13 — Source Data [file 41467_2021_25556_MOESM13_ESM.zip › Supplementary Software/Controls/Control-01-Sample/20.bmp]
